# Breastfeeding strategies following nurslings’ hospitalization: a scoping review*

**DOI:** 10.1590/1518-8345.7800.4674

**Published:** 2025-11-10

**Authors:** Socorro Alana Ramalho Rocha, Eliane Cristina da Silva Buck, Cintia Bezerra Almeida Costa, Smalyanna Sgren da Costa Andrade

**Affiliations:** 1 Faculdade de Enfermagem e Medicina Nova Esperança, João Pessoa, PB, Brazil Faculdade de Enfermagem e Medicina Nova Esperança PB João Pessoa Brazil; 2 Universidade Federal da Paraíba, Departamento de Enfermagem, João Pessoa, PB, Brazil Universidade Federal da Paraíba Departamento de Enfermagem PB João Pessoa Brazil

**Keywords:** Breastfeeding, Patient Discharge, Nursing Care, Health Strategies, Primary Health Care, Lactation

## Abstract

to map strategies aimed at promoting and maintaining breastfeeding following nurslings’ hospitalization.

a scoping review guided by the recommendations of the Joanna Briggs Institute Reviewer’s Manual*.* The search was carried out in seven databases and in repositories of theses and dissertations. The selection of studies was conducted on a web application by two independent blind reviewers. The data were analyzed descriptively.

in total, 1,325 publications were obtained, of which 29 were included in the study. The following were identified as health strategies associated with breastfeeding: education and guidance, support and follow-up, clinical and technical interventions, complementary therapies, technology and innovation, environment and physical conditions, nutrition and hydration, protocol and guidelines.

the evidence reinforces that multifaceted strategies are essential post-discharge in order to strengthen self-management and sustain breastfeeding beyond the hospital setting. Primary Health Care can play an important role in care continuity.

## Highlights

**(1)** With evidence-based techniques, breastfeeding can be re-established. **(2)** Family and professional support is essential for maintaining breastfeeding. **(3)** Education and social support increase the duration and exclusivity of breastfeeding. **(4)** Technologies increase access to breastfeeding information and support. **(5)** Complementary interventions help to optimize milk production.

## Introduction

Newborns and nurslings are more vulnerable to illness due to their immune immaturity. Exclusive breastfeeding (EBF) is essential for strengthening immunity and preventing illness, but separation from the mother during hospitalization, associated with prematurity, can lead to early weaning, thus affecting child development and increasing the risk from hospitalizations^(1)^.

The mother’s presence during hospitalization is crucial, and practices such as skin-to-skin contact, rooming-in and educational support for breastfeeding (BF) are recommended, as evidenced by a systematic review^(2)^. In addition, sucking stimulation is essential, with the support of trained professionals and appropriate protocols^(3)^. In this context, the Academy of Breastefeeding Medicine (ABM) develops guidelines for successful breastfeeding, such as clinical protocol no. 35, which supports breastfeeding during hospitalization^(4)^.

Therefore, the use of innovative approaches and health tools can strengthen women’s learning, promoting breastfeeding and encouraging preventive behaviors^(5)^. The technologies involved, which include management, educational and care interventions, are essential for health promotion, prevention and care^(6)^. In maternal and child care, educational materials help provide guidance on breastfeeding, thus stimulating family autonomy and supporting maternal self-efficacy^(7)^.

A clinical trial with 112 pregnant women showed that the use of a serial album in the intervention group significantly increased breastfeeding rates, with the likelihood of success being twofold as compared to that in the control group (p<0.001)^(8)^. In turn, the study on the development and application of the leaflet “Every woman can breastfeed!” showed that this educational technology promoted maternal empowerment and the sharing of knowledge with puerperal women and their companions, thus strengthening self-efficacy for breastfeeding^(9)^.

Nurses play an essential role in promoting and supporting breastfeeding, with technical guidance and emotional support, especially in Primary Health Care (PHC), which coordinates care in the Health Care Network^(10)^. With a focus on child health, its approach prioritizes bonds, maternal experiences and the use of soft technologies^(11)^, empowering women, families and communities in the home environment. Such integrated care can reduce hospital admissions and strengthen public policies^(6)^_,_ thus consolidating PHC as a key point for encouraging breastfeeding.

In view of the above, the objective of this study is to map out strategies aimed at promoting and maintaining breastfeeding after a nursling has been admitted to hospital. This review is based on the possibility of gathering information to support the construction of future soft-hard technologies aimed at protecting and providing basic support for breastfeeding in the home environment.

## Method

### Study design

This scoping review was carried out following the methodology of the Joanna Briggs Institute (JBI)^(12)^, concomitantly with the recommendations from the Preferred Reporting Items for Systematic Reviews and Meta-Analyses (PRISMA-ScR) checklist^(13)^. The protocol was registered on the Open Science Framework (OSF) platform, which can be accessed via the following link: https://doi.org/10.17605/OSF.IO/XJ374

In order to carry out the review, the following stages were considered: Phase 1 - Eligibility criteria; Phase 2 - Sources of information and literature search; Phase 3 - Selection of sources of evidence; Phase 4 - Data extraction; and Phase 5: Data analysis and presentation.

### Phase 1 - Eligibility criteria

The acronym PCC (population, concept and context) was used to formulate the study objective: P (population) = nurslings; C (concept) = breastfeeding; C (context) = post-hospitalization. The study’s guiding question was: what scientific production is there on strategies to promote and maintain breastfeeding following nurslings’ hospitalization?

Complete studies aligned with the topic were considered, covering various methodological designs, both published and unpublished (gray literature), with no language restrictions. As for the period of publication, the interval from 2012 to 2024 was established, with 2012 being the year of approval of the global target related to child nutrition, stipulated by WHO/PAHO, through the World Health Assembly, with a view to promoting a 50% increase in the EBF rate by 2025^(14)^. This was considered a political milestone with a major impact on the issue.

### Phase 2 - Information sources and literature search

In order to identify the relevant studies, we consulted the periodical databases of the Nursing Database (BDENF), Latin American and Caribbean Health Sciences Literature (LILACS), accessed via the Virtual Health Library (VHL) platform, as well as the National Library of Medicine (PubMed), Scientific Electronic Library Online (SciELO), Cumulative Index Nursing Allied Health Literature (CINAHL), Cochrane Library and Scopus.

In addition, a survey was carried out in additional sources, such as Google Scholar, the CAPES Theses and Dissertations Catalog and the Brazilian Digital Library of Theses and Dissertations (BDTD). A manual search was also carried out on the list of references of the selected studies.

In order to obtain a comprehensive overview of the current state of knowledge, a four-stage search strategy was developed, following the guidelines of the JBI manual for scoping reviews^(12)^. Firstly, an initial search was conducted in the BDENF and PubMed databases, using DeCS-MeSH terms and index terms, to examine the titles and abstracts of the articles found and identify terms to be added to the search strategy. The research tactic was then adapted for the other databases, considering their individual particularities.

Subsequently, there was a search of gray literature sources, complementing the findings of the productions indexed in the databases, in order to encourage the incorporation of information related to technical management in BF. In addition, a manual search was carried out on the list of references of the selected studies to ensure that the information was up to date.

The search process incorporated controlled terms and keywords pertinent to the components of the PCC strategy, combined with the Boolean operators AND and OR. The search approach, covering all the keywords and indexing terms identified, was duly adapted for each source of information consulted, as shown in Figure 1 below:

**Figure 1-:** Database search strategy. Campina Grande, PB, Brazil, 2024

BDENF	(ti:( *aleitamento materno* )) OR ( *aleitamento* ) OR ( *amamentação* ) OR ( *alimentação ao peito* ) AND ( *alta do hospital* ) AND ( *cuidados de enfermagem* ) OR ( *cuidados primários de enfermagem* ) AND ( *atenção primária à saúde* ) OR ( *atenção básica* ); (ti:( *aleitamento materno* )) OR ( *amamentação* ) OR ( *alimentação ao peito* ) AND ( *estratégias* ) AND ( *cuidados de enfermagem* ) OR ( *cuidados primários de enfermagem* ) AND ( *atenção primária à saúde* ) OR ( *atenção básica* )
PubMed	((((((((breastfeeding[Title]) OR (breast feeding[Title])) AND (patient discharge[MeSH Terms])) OR (hospitalization[MeSH Terms])) AND (nursing care[MeSH Terms])) OR (nursing care plan[MeSH Terms])) AND (health strategies[All Fields])) OR (local health strategies[All Fields])) AND (primary health care[MeSH Terms])) OR (care, primary health[MeSH Terms])
LILACS	(ti:( *aleitamento materno* )) OR ( *aleitamento* ) OR ( *amamentação* ) OR ( *alimentação ao peito* ) AND ( *alta do hospital* ) AND ( *cuidados de enfermagem* ) OR ( *cuidados primários de enfermagem* ) AND ( *atenção primária à saúde* ) OR ( *atenção básica* ); (ti:( *aleitamento materno* )) OR ( *amamentação* ) OR ( *alimentação ao peito* ) AND ( *estratégias* ) AND ( *cuidados de enfermagem* ) OR ( *cuidados primários de enfermagem* ) AND ( *atenção primária à saúde* ) OR ( *atenção básica* )
SciELO	(ti:( *aleitamento materno* )) OR ( *amamentação* ) AND ( *cuidados de enfermagem* ) AND ( *atenção primária à saúde* ) OR ( *atenção básica* )
Scopus	(((TITLE(Breast) AND (TITLE(Feed*) OR TITLE(Breastfeed*) OR TITLE(Breastfeeding)) AND (TITLE-ABS(Patient discharge*) OR TITLE-ABS(Infant discharge) AND (TITLE-ABS(«Nursing care») AND TITLE-ABS(Strategies) OR TITLE-ABS(«Associated Factor»)) AND TITLE-ABS(Primary health care)))
CINAHL	TI ((breastfeeding OR breast-feeding OR infant feeding OR lactation OR lactating) AND (hospital discharge of the infant) AND (strategies OR resources OR methods OR techniques) AND (nursing care OR nursing interventions) AND (primary health care))
Cochrane Library	“breastfeeding” in Title Abstract Keyword AND “strategies” in All Text AND “primary health care” in All Text - with Publication Year from 2012 to 2024, in Trials (Word variations have been searched)
*Catálogo de Teses e Dissertações - CAPES*	“ *aleitamento materno* ” OR “ *amamentação* ” AND “ *atenção primária à saúde* ” AND “ *cuidados de enfermagem* ”
BDTD*	“( *Título:aleitamento materno* AND *Todos os campos:alta hospitalar* AND *Todos os campos:atenção primária à saúde* )”

*BDTD = Brazilian Digital Library of Theses and Dissertations

### Phase 3 - Selecting sources of evidence

The studies obtained from the search of the aforementioned databases were transferred to the EndNote reference manager in order to automatically eliminate duplicate studies. They were then exported to the Rayyan software, where the stages for study exclusion were performed, beginning with an analysis of the titles and abstracts, followed by a complete reading. These steps were carried out independently by two reviewers, without any divergence regarding the exclusion of articles, which eliminated the need to consult a third reviewer.

### Phase 4 - Data extraction

The data extraction phase was carried out with the purpose of organizing, analyzing and interpreting the information present in the selected studies, according to the established objective. For this procedure, a data extraction form was structured, covering information such as identification of the journal/source, author, year, country of origin of the study, objectives, population and sample, methodological design, main study outcomes and the authors’ conclusion.

### Phase 5: Data analysis and presentation

Data analysis involved drawing up a synthesis of the findings, in line with the objectives of the review, and discussing the implications of the results. Based on the information extracted, a descriptive analysis was carried out and the results were organized in a table with the main characteristics of the studies included.

The studies were classified into five levels of evidence: Level 1 covers systematic reviews and reliable randomized clinical trials; Level 2, cohort studies and “outcomes” research; Level 3, case-control studies; Level 4, case series and observational studies without controls; and Level 5, expert opinions without critical evaluation^(15)^. This classification organizes the quality of the evidence, guiding decisions based on the most reliable data.

### Ethical aspects

As this was a scoping review, it was not necessary to request approval from the Research Ethics Committee. We also declare that there is no conflict of interest.

## Results

**Figure 2-:**
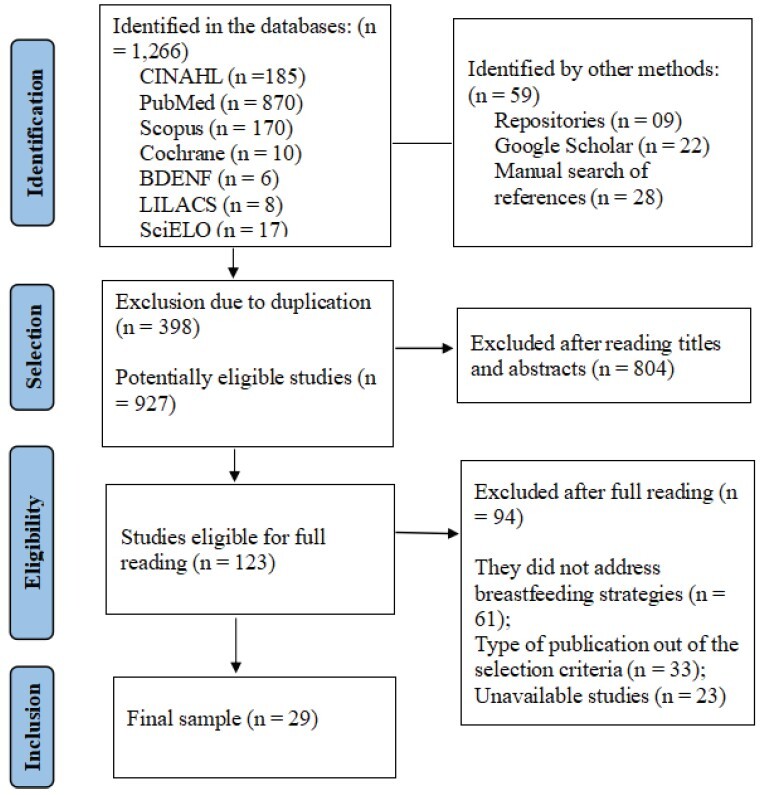
Flowchart of the selection process for the studies included in the review. Campina Grande, PB, Brazil, 2024

A total of 1,325 studies (articles, documents, theses and dissertations) were identified, of which 398 were excluded due to duplication, using the EndNote program. After analyzing the titles and abstracts, 123 studies were selected for full reading, of which 29 formed the final sample. The Preferred Reporting Items for Systematic Review and Meta-Analyses (PRISMA) was used to describe the searches and select the studies, as illustrated in Figure 2 below

Note: Adapted according to the guidelines of the Preferred Reporting Items for Systematic Review and Meta-Analyses (PRISMA-ScR)^(13)^.

As for the continents, the studies were mostly distributed in South America (41.38%), followed by North America (31.03%), Asia (13.79%), Europe (6.9%), Oceania (3.45%) and Central America (3.45%).

The characterization of the studies identified is shown below, considering aspects such as authorship, year and place of publication, as well as design and level of evidence, as shown in Figure 3.

**Figure 3-:** Characterization of the studies included in the review. Campina Grande, PB, Brazil, 2024

**Article**	**Main author**	**Year**	**Country**	**Type of study**	Level of evidence*
1	Iopp, et al.	2023	Brazil	Descriptive and cross-sectional	2c
2	Martins, et al	2024	Brazil	Descriptive and qualitative	2c
3	Sobral, et al	2023	Brazil	Retrospective and mixed approach	2c
4	Montoya, et al.	2020	Colombia	Descriptive and cross-sectional	2c
5	Lojander, et al	2024	Finland	Non-experimental correlational study	5
6	Kwan, et al.	2021	Malaysia	Scope review	4
7	Top, et al.	2022	Turkey	Descriptive and cross-sectional	2c
8	Luz, et al.	2018	Brazil	Open, prospective cohort study	2b
9	Araújo, et al	2023	Brazil	Integrative review	4
10	Oswaldo Cruz Foundation	2018	Brazil	Good practice protocol	5
11	Cordell, et al.	2020	USA	Systematic review	1a
12	Wood, et al.	2016	USA	Systematic review	1a
13	Santos, et al.	2012	Brazil	Integrative review	4
14	Zanlorenzi GB	2022	Brazil	Methodological study	5
15	Dahl L	2015	USA	Book chapter	5
16	Álvarez-Peña, et al.	2023	Mexico	Descriptive and qualitative	2c
17	Levene, et al.	2024	United Kingdom	Systematic review and meta-analysis	1a
18	Laili	2021	Indonesia	Systematic review	2a
19	Ministry of Health (BR)	2015	Brazil	Manual of the Brazilian Ministry of Health	5
20	Boersma, et al.	2017	Canada	Good practice protocol	5
21	Parker	2013	USA	Integrative review	4
22	Ahmed, et al.	2016	USA	Randomized controlled clinical trial	1b
23	Düzgün, et al.	2020	Turkey	Systematic review and meta-analysis	1a
24	Noble, et al.	2018	USA	Good practice protocol	5
25	Brodribb	2018	USA	Clinical protocol	5
26	Hoyt-Austin, et al.	2022	USA	Clinical protocol	5
27	Wright A.	2022	Australia	Good practice protocol	5
28	Rodrigues, et al.	2013	Brazil	Integrative review	4
29	Ministry of Health (BR)	2013	Brazil	Government document	5

*Classification of levels of evidence according to the Oxford Centre for Evidence-Based Medicine (2009)^(15)^

Regarding the methodological design, review studies predominated (34.48%), including scoping, integrative and systematic studies, followed by protocols and guidelines (27.59%), cross-sectional studies (13.79%) and qualitative cross-sectional studies (6.9%). Finally, there was only one article for a cohort study (3.45%), randomized clinical trial (3.45%), non-experimental correlational study (3.45%), thesis (3.45%) and book chapter (3.45%). As for the period of publication, the majority were published between 2018 and 2024 (68.97%), followed by the period between 2012 and 2017 (31.03%).

The table below summarizes the main characteristics of the studies included in the scoping review, highlighting key aspects such as objectives, main outcomes, conclusions and the strategies obtained. The detailed presentation of the studies makes it possible to assess the relevance and quality of the approaches investigated, facilitating critical analysis and directing interpretations, as shown in Figure 4:

**Figure 4-:** Description of the studies included in the scoping review. Campina Grande, PB, Brazil, 2024

ID*	**Objectives**	**Main outcomes and conclusion**	**Strategies**
A ^†^ 1	To learn about nurses’ actions in supporting BF ^‡^ .	Support reduces early weaning and addresses risks.	Guidance on massage, milk extraction, care for premature babies and twins.
A ^†^ 2	To describe the experience of promoting BF ^‡^ in a UBS ^§^ .	Health indicators have improved. Challenges include staff turnover.	Group activities, information materials and team support.
A ^†^ 3	To analyze translactation and relactation when resuming BF ^‡^ .	78.6% effectiveness in reversing weaning.	Translactation, relactation, stimulation and breast milking.
A ^†^ 4	To identify the causes of abandonment and success in relactation.	Effective relactation for premature babies, with continuous support.	Combination of BF ^‡^ + supplementation and the use of relactators.
A ^†^ 5	To analyze the exclusivity of BF ^‡^ after hospital discharge.	Rapid support improves BF ^‡^ in primiparous women.	To restrict supplements, digital support and home visits.
A ^†^ 6	To examine the galactagogue activity of plants.	Fenugreek and milk thistle have been shown to be effective. Further research is recommended.	Supervised use of galactagogues.
A ^†^ 7	To determine methods to increase breast milk.	Traditional and modern practices strengthen trust.	Effective emptying, cultural approaches and training.
A ^†^ 8	To evaluate exclusive BF ^‡^ in post-discharge premature infants.	Rates below those recommended by WHO ^‖^ .	Public policies, continuous care and post-discharge support.
A ^†^ 9	To analyze evidence on breast milk production.	Methods such as acupuncture and relactation are effective at 72.22%.	Use of medicines, herbal medicines and educational protocols.
A ^†^ 10	To maintain lactation and establish effective BF ^‡^ .	Home support and techniques such as translactation are essential.	Translaction, weight monitoring and latching adjustment.
A ^†^ 11	To analyze the impact of social support on BF ^‡^ .	Social support and educational interventions prolong BF ^‡^ .	Support groups, community programs and communication channels.
A ^†^ 12	To evaluate interventions on BF ^‡^ .	Education on behavior and signs improves the perception of milk insufficiency.	Educational approach and self-management sessions.
A ^†^ 13	To learn about practices for BF ^‡^ premature infants in the ICU ^¶^ .	Support from the professional and family network is essential for success.	Skin-to-skin contact, moisturizing and complementary therapies.
A ^†^ 14	To develop a nursing protocol on BF ^‡^ in PHC**.	Continuous training and the use of technology improve indicators.	Audiovisual and digital education with a cultural approach.
A ^†^ 15	To address breastfeeding problems.	Inefficient extraction reduces production. Proper techniques are essential.	Comfortable vacuum pumping and relaxation techniques.
A ^†^ 16	To share experiences on relactation.	It strengthens the mother-baby bond and quality of life.	Relactation with technical and emotional support.
A ^†^ 17	Evaluate the impact of relaxation on BF ^‡^ .	Relaxation increases milk production and reduces maternal anxiety.	Music therapy and relaxation practices.
A ^†^ 18	To learn about how to increase milk production.	Combined methods improve lactation and reduce stress.	Massage, acupressure, fenugreek and positive affirmations.
A ^†^ 19	To promote BF ^‡^ with comprehensive child health care.	Family support and a balanced diet overcome difficulties.	Community involvement, latching techniques and adequate calorie intake.
A ^†^ 20	To support evidence-based clinical practice.	Adjustments ensure the proper transfer of milk and nutrition.	Breast compressions and alternate feeding.
A ^†^ 21	To analyze milk expression strategies in premature infants.	Guidelines should emphasize early onset and more frequent expression.	Suitable pumps and specific training for mothers.
A ^†^ 22	To determine the impact of the interactive system on BF ^‡^ .	Online monitoring improves BF ^‡^ in underprivileged populations.	Web interventions and expert support.
A ^†^ 23	To investigate the effect of music therapy on BF ^‡^ .	Music reduces stress and increases milk production.	Encouraging musical choice and regular sessions.
A ^†^ 24	To guide post-discharge care from the neonatal ICU ^¶^_._	An effective transition requires a joint plan and maternal adjustments.	Latch-on monitoring, triple feeding and breastfeeding plan.
A ^†^ 25	To create protocols for BF ^‡^ problems.	Galactagogues should be used under medical supervision.	Assessment of hypolactation and effective breast drainage.
A ^†^ 26	To provide post-discharge BF ^‡^ support.	Pain care and ongoing support improve indicators.	Home visits and remote support from professionals.
A ^†^ 27	To provide information on BF ^‡^ to professionals.	Optimized production improves confidence and reduces supplementation.	Night feeds, effective extraction and adequate rest.
A ^†^ 28	To evaluate factors associated with BF ^‡^ in premature infants.	Milking and the mother-baby bond are essential for premature babies.	Cup use, stress reduction and maternal education.
A ^†^ 29	To promote exclusive BF and healthy complementary feeding.	The Breastfeeding Brazil Strategy qualifies professionals and reduces inadequate practices.	Qualifying, monitoring and encouraging breastfeeding.

*ID = Identification of production; ^†^A1-^†^A29 = Productions from 1 to 29; ^‡^BF = Breastfeeding; ^§^UBS = Basic Health Unit; ^‖^WHO = World Health Organization; ^¶^ICU = Intensive Care Unit; **PHC = Primary Health Care

For analysis purposes, the included studies were organized into four thematic categories: educational and social approaches to promoting breastfeeding (31.0%), family, community and professional support for breastfeeding (34.5%), techniques for resuming breastfeeding (24.1%) and complementary interventions to optimize milk production (34.5%). Considering the multifactorial nature of the interventions, the same study could be classified in more than one category.

Below are the main strategies identified in the included studies, distributed according to the four thematic categories described above. These strategies include evidence-based actions aimed at continuous breastfeeding support, as illustrated in Figure 5:

**Figure 5-:** Strategies for maintaining BF identified in the scoping review. Campina Grande, PB, Brazil, 2024

Educational and social approaches to promoting breastfeeding
A2, A11: Educational actions, social support, group activities and information materials.
A13: Creating a calm environment and skin-to-skin contact.
A14: Nursing protocol with videos and illustrations.
A1, A24: Guidance on proper latch-on, risks of formula and artificial nipples.
A10: Maintaining proper latch-on and observing coordination between sucking and breathing.
A5: The use of digital alternatives to improve maternal satisfaction.
A22: Web-based intervention with monitoring and notifications.
Family, community and professional support for breastfeeding
A1, A8: Continuous multi-professional support.
A10, A12: Home monitoring and weight gain monitoring.
A19, A29: Continuous support from PHC* professionals.
A4, A19, A26: Continuous support from family, community and professionals.
A14: Development of a nursing protocol for breastfeeding management.
A25: Development of clinical protocols to manage medical problems related to breastfeeding.
Techniques for resuming breastfeeding
A3, A4: Translactation and relactation techniques.
A16: Frequent stimulation and skin-to-skin contact during relactation.
A15: Combination of manual stimulation and electric pumping.
A20: Alternating breasts during feeding (switch nursing) to stimulate the ejection reflex.
A4, A28: The use of devices to support BF (cup, relactator).
A21: Early start and increased frequency of milk expression.
A21, A28: Skin-to-skin contact to improve milk production.
Complementary interventions to optimize milk production
A9, A13: Massage and acupuncture to increase milk production.
A18: Combination of acupressure and positive affirmations.
A17: Relaxation interventions to improve lactation and maternal well-being.
A23: Musical intervention to optimize milk production.
A6: The use of galactagogue plants such as fenugreek.
A7, A9: The use of medicines and herbal medicines.
A25: The use of domperidone and metoclopramide as galactagogues.
A19, A27: Increased calorie and fluid intake; balanced diet.

A = Production; *PHC = Primary Health Care

Promoting breastfeeding faces interconnected challenges that require effective strategies. Misinformation can be tackled with educational actions, while nipple confusion, associated with the use of pacifiers and bottles, requires guidance on safe alternatives. Stress and sleep deprivation, in turn, require support networks and encouragement for self-care. Hypogalactia, which may be related to these factors, can be addressed with frequent breast stimulation, skin-to-skin contact and appropriate devices, combined with technical and emotional support.

## Discussion

Based on the systematized results, it can be seen that the strategies aimed at promoting, supporting and resuming BF involve different approaches, grouped into four thematic categories for analysis purposes. Each of these categories is discussed below, in the light of the available evidence and the settings in which the interventions were applied.

### Techniques for resuming breastfeeding

Several studies have shown the effectiveness of relactation and translactation practices in re-establishing breastfeeding, especially in cases of early weaning and in premature newborns. With the right support, relactation has been shown to be effective, helping to produce breast milk, especially in low-birth weight infants^(16)^. In this context, one study showed that 78.6% of infants resumed breastfeeding after implementing these techniques^(17)^. In addition, some protocols, such as ABM Clinical Protocol #12, guide the transition of premature babies from the NICU to home, promoting breastfeeding^(18)^, while ABM Clinical Protocol #9 addresses supplementation with human milk or formula and the use of assistive devices^(19)^.

Furthermore, a study revealed that the early onset of milk expression, the increased frequency of extraction sessions and skin-to-skin contact are decisive factors in optimizing milk production in mothers of very low-birth-weight babies, also highlighting the importance of access to adequate pumps and the training of health professionals to support mothers^(20)^. In this context, breast compressions are recommended to improve milk transfer. Switching breasts during feeding (switch nursing) has also been identified as an effective technique for stimulating multiple milk letdowns, ensuring better nutrition for the baby and a more positive experience for the mother^(21)^.

### Family, community and professional support for breastfeeding

Continued support from professionals and family members is essential for maintaining breastfeeding, especially after discharge from hospital^(22)^. Studies carried out in the United States show that educational and social interventions can increase the duration and exclusivity of breastfeeding^(23)^ by overcoming barriers such as the perception of insufficient milk^(24)^. In Brazil, prenatal education has been shown to be effective in improving health indicators related to breastfeeding^(25)^.

Ministerial documents emphasize the importance of multi-professional support and family and community participation for successful breastfeeding up to the age of two^(26)^. This support should continue after hospital discharge, with community follow-up and regular consultations^(27)^. ABM Clinical Protocol #2 reinforces family-centered follow-up^(28)^, and care models with training and community support have shown good results in the Unified Health System (SUS), helping to prevent early weaning^(29)^.

### Complementary interventions to optimize milk production

A review of clinical studies in Malaysia evaluated the use of galactagogue plants, such as fenugreek and milk thistle, which showed positive effects on breast milk production^(30)^ and are natural and effective alternatives for mothers with lactation difficulties^(31)^. In addition, non-pharmacological practices, such as acupuncture and music therapy, have also shown potential to increase milk production and improve mothers’ emotional well-being, despite variable results^(32)^. The combination of these interventions, along with techniques such as breast massage, acupressure and the use of galactagogues, can promote lactation and reduce maternal stress^(33)^.

Acupuncture has shown potential to increase breast milk production by stimulating hormones such as oxytocin and prolactin. A systematic review indicated that many mothers who underwent acupuncture sessions reported a significant increase in the amount of milk. In addition, this technique helps to reduce stress and pain by promoting relaxation, which facilitates breastfeeding. Although widely used in Asia, rigorous research to standardize protocols and evaluate its effectiveness still lacks, but evidence suggests that it can be useful for mothers with lactation difficulties^(34)^.

Music therapy has shown positive effects in promoting BF, especially when mothers listen to relaxing music while expressing milk, with 30-minute sessions repeated at least 10 times, which can increase milk production and reduce stress^(35)^. It also helps to reduce anxiety and improves mothers’ mental health, which are important factors for successful breastfeeding. Although the evidence on its effects on babies’ sleep and on reducing cortisol in milk is limited, the practice is well accepted, risk-free and affordable for mothers^(36)^.

### Educational and social approaches to promoting breastfeeding

Educational interventions and ongoing social support increase the duration and exclusivity of breastfeeding by addressing modifiable causes such as stress and lack of knowledge about milk production^(25)^. Group activities, information materials and on-call support from health teams are essential for successful breastfeeding^(29)^ as well as frequent contact with mothers and their support networks^(23)^.

The use of digital technologies has been highlighted as an essential resource in breastfeeding support, especially among vulnerable populations, offering access to information and real-time guidance for mothers with limited access to onsite^(22)^. These tools expand breastfeeding support by providing quality data and personalized help^(32)^. Online support groups, direct channels with experts and monitoring platforms have shown effectiveness in resolving challenges, prolonging breastfeeding and creating a favorable environment^(37)^.

In addition, nursing training must be improved to strengthen support for BF, emphasizing the continuous training of professionals. This involves the use of digital resources and the integration of innovative technologies that help address cultural and social challenges related to breastfeeding^(29)^.

The limitations in this study were the small number of studies carried out specifically in Primary Health Care. Strategies with potential for application at this care level were included, considering the role of PHC in care continuity. In addition, some articles were excluded because they were not available in full. To minimize bias, the search was expanded to several databases, including protocols and related documents. The classification of the level of evidence was not used as a criterion for accepting or rejecting strategies, as it was not part of the methodological scope. Further research is, therefore, recommended to explore the determinants identified and their applicability to real outcomes.

## Conclusion

The scoping review identified different strategies for maintaining and resuming BF after hospitalization, highlighting educational actions, multi-professional and family support, evidence-based interventions, complementary therapies and the use of technologies to increase access to information and support. It also highlights the importance of favorable settings and guidelines to guide professional practice in the home context.

Further research into the effectiveness of these interventions is recommended, with a focus on the use of technologies in post-discharge follow-up, the standardization of techniques and the evaluation of impacts on BF continuity. These results can support public policies and improve maternal and child care in Primary Health Care.

Lastly, there is a need for interdisciplinary actions that increase access to and resolution of post-discharge care, integrating these measures into daily care and strengthening breastfeeding promotion and support.

## Data Availability

All data generated or analysed during this study are included in this published article.
