# Supplementary material for: Breastfeeding strategies following nurslings’ hospitalization: a scoping review
Source: Rev Lat Am Enfermagem. 2025 Nov 10;33:e4674. doi: 10.1590/1518-8345.7800.4674 (PMC12610927; doi:10.1590/1518-8345.7800.4674)
Supplement: Translation

# Translation: Estratégias para o aleitamento materno após a internação hospitalar de lactentes: uma revisão de escopo*

**DOI:** 10.1590/1518-8345.7800.4675

**Authors:** Socorro Alana Ramalho Rocha, Eliane Cristina da Silva Buck, Cintia Bezerra Almeida Costa, Smalyanna Sgren da Costa Andrade

**Affiliations:** 1 Faculdade de Enfermagem e Medicina Nova Esperança, João Pessoa, PB, Brasil Faculdade de Enfermagem e Medicina Nova Esperança PB João Pessoa Brasil; 2 Universidade Federal da Paraíba, Departamento de Enfermagem, João Pessoa, PB, Brasil Universidade Federal da Paraíba Departamento de Enfermagem PB João Pessoa Brasil

**Keywords:** Aleitamento Materno, Alta do Paciente, Cuidados de Enfermagem, Estratégias de Saúde, Atenção Primária à Saúde, Lactação

## Abstract

mapear estratégias voltadas à promoção e à manutenção do aleitamento materno após a internação hospitalar do lactente.

revisão de escopo guiada pelas recomendações do*Joanna Briggs Institute Reviewer’s Manual.* A busca foi realizada em sete bases de dados e em repositórios de teses e dissertações. A seleção dos estudos foi conduzida em um aplicativo da *web* por dois revisores independentes e às cegas. A análise dos dados foi realizada de forma descritiva.

obtiveram-se 1.325 publicações, das quais 29 foram incluídas no estudo. Foram identificadas como estratégias em saúde associadas à amamentação: educação e orientação, suporte e acompanhamento, intervenções clínicas e técnicas, terapias complementares, tecnologia e inovação, ambiente e condições físicas, nutrição e hidratação, protocolo e diretrizes.

as evidências reforçam que estratégias multifacetadas são essenciais no pós-alta para fortalecer a autogestão e sustentar a amamentação além do ambiente hospitalar. A Atenção Primária à Saúde pode desempenhar papel importante na continuidade do cuidado.

## Destaques

**(1)** Com técnicas baseadas em evidências, é possível restabelecer a amamentação. **(2)** O suporte familiar e profissional é essencial para a manutenção do aleitamento. **(3)** Educação e apoio social aumentam a duração e a exclusividade da amamentação. **(4)** Tecnologias ampliam o acesso a informações e suporte à amamentação. **(5)** Intervenções complementares ajudam a otimizar a produção de leite.

## Introdução

Recém-nascidos e lactentes possuem maior vulnerabilidade ao adoecimento devido à imaturidade imunológica. O aleitamento materno exclusivo (AME) é essencial para fortalecer a imunidade e prevenir doenças, mas a separação da mãe durante a internação hospitalar, relacionada à condição de prematuridade, pode levar ao desmame precoce, afetando o desenvolvimento infantil e aumentando o risco de hospitalizações^(1)^.

A presença da mãe durante a internação é crucial, sendo recomendadas práticas como o contato pele a pele, alojamento conjunto e suporte educativo ao aleitamento materno (AM), conforme evidenciado por uma revisão sistemática^(2)^. Além disso, é essencial a estimulação da sucção, com o apoio de profissionais capacitados e protocolos adequados^(3)^. Nesse contexto, a Academia de Medicina da Amamentação (*Academy of Breastefeeding Medicine* - ABM) desenvolve diretrizes para o sucesso da amamentação, como o protocolo clínico nº 35, que apoia o aleitamento durante a internação^(4)^.

Por conseguinte, o uso de abordagens inovadoras e ferramentas em saúde pode fortalecer a aprendizagem das mulheres, promovendo o AM e incentivando comportamentos preventivos^(5)^. As tecnologias envolvidas, que incluem intervenções gerenciais, educacionais e assistenciais, são essenciais para a promoção, prevenção e cuidados em saúde^(6)^. Na área materno-infantil, materiais didático-pedagógicos auxiliam na orientação sobre o AM, estimulando a autonomia das famílias e apoiando a autoeficácia materna^(7)^.

Um ensaio clínico com 112 gestantes mostrou que o uso do álbum seriado no grupo-intervenção aumentou significativamente as taxas de AME, com probabilidade duas vezes maior de sucesso em relação ao grupo-controle (p<0,001)^(8)^. Por sua vez, o estudo sobre o desenvolvimento e aplicação do *folder* “Toda mulher é capaz de amamentar!” apontou que essa tecnologia educativa promoveu o empoderamento materno e o compartilhamento de conhecimento com puérperas e seus acompanhantes, fortalecendo a autoeficácia para amamentar^(9)^.

O enfermeiro desempenha papel essencial na promoção e suporte ao AM, com orientação técnica e apoio emocional, especialmente na Atenção Primária à Saúde (APS), que coordena o cuidado na Rede de Atenção^(10)^. Com foco na saúde infantil, sua abordagem prioriza vínculos, experiências maternas e o uso de tecnologias leves^(11)^, empoderando mulheres, famílias e comunidades no ambiente domiciliar. Esse cuidado integrado pode reduzir internações hospitalares e fortalecer políticas públicas^(6)^, consolidando a APS como ponto-chave para o incentivo à amamentação.

Diante do exposto, o objetivo do estudo é mapear estratégias voltadas à promoção e manutenção do AM materno após a internação hospitalar do lactente. Esta revisão se respalda na possibilidade de reunir uma coletânea de informações que fundamentem a construção de futuras tecnologias leve-duras, voltadas à proteção e oferta de um suporte básico ao seguimento do AM em ambiente domiciliar.

## Método

### Delineamento do estudo

Esta revisão de escopo foi realizada seguindo a metodologia do Instituto Joanna Briggs (JBI)^(12)^, concomitantemente com as recomendações do *checklist Preferred Reporting Items for Systematic Reviews and Meta-Analyses* (PRISMA-ScR)^(13)^. O protocolo foi registrado na plataforma *Open Science Framework* (OSF), com acesso por meio do link: https://doi.org/10.17605/OSF.IO/XJ374

Para a realização da revisão foram consideradas as seguintes etapas: Fase 1 - Critérios de elegibilidade; Fase 2 - Fontes de informação e busca na literatura; Fase 3 - Seleção de fontes de evidência; Fase 4 - Extração de dados; e Fase 5: Análise e apresentação dos dados.

### Fase 1 - Critérios de elegibilidade

Utilizou-se o acrônimo PCC (população, conceito e contexto) para a formulação do objetivo do estudo: P (população) = lactentes; C (conceito) = aleitamento materno; C (contexto) = pós-hospitalização. A questão norteadora do estudo foi: quais são as produções científicas acerca das estratégias voltadas à promoção e manutenção do AM após a internação hospitalar de lactentes?

Foram considerados estudos completos alinhados à temática, abrangendo diversos desenhos metodológicos, tanto publicados quanto não publicados (literatura cinzenta), sem restrição de idioma. Quanto ao período de publicação, foi estabelecido o intervalo de 2012 a 2024, sendo 2012 o ano de aprovação da meta global relacionada à nutrição infantil, estipulada pela OMS/OPAS, por meio da Assembleia Mundial de Saúde, com vistas a promover o aumento, em 50%, da taxa de AME até 2025^(14)^. Esse foi considerado um marco político de grande impacto voltado à temática.

### Fase 2 - Fontes de informação e busca na literatura

Para a identificação dos estudos relevantes, foram consultados os bancos de dados de periódicos da Base de Dados de Enfermagem (BDENF), Literatura Latino-americana e do Caribe em Ciências da Saúde (LILACS), acessadas via plataforma da Biblioteca Virtual em Saúde (BVS), além da *National Library of Medicine* (*PubMed*), *Scientific Electronic Library Online* (SciELO), *Cummulative Index Nursing Allied Health Literature* (CINAHL), *Cochrane Library* e *Scopus*.

Outrossim, realizou-se um levantamento em fontes adicionais, como o *Google Scholar*, Catálogo de Teses e Dissertações da CAPES e Biblioteca Digital Brasileira de Teses e Dissertações (BDTD). Além disso, foi realizada uma busca manual na lista de referências dos estudos selecionados.

Com a finalidade de obter uma visão abrangente do atual estado do conhecimento, foi desenvolvida uma estratégia de busca em quatro etapas, seguindo as diretrizes do manual para revisões de escopo do JBI^(12)^. Primeiramente, conduziu-se uma pesquisa inicial nas bases BDENF e *PubMed*, utilizando termos DeCS-MeSH e termos índices, para examinar os títulos e resumos dos artigos encontrados e identificar termos a serem adicionados à estratégia de busca. Em seguida, a tática de investigação foi adaptada para as demais bases de dados, considerando suas particularidades individuais.

Posteriormente, houve uma busca em fontes da literatura cinzenta complementando os achados das produções indexadas nas bases de dados, no sentido de favorecer a incorporação de informações relacionadas ao manejo técnico no AM. Ademais, realizou-se uma busca manual na lista de referências dos estudos selecionados, para assegurar a atualização das informações.

O processo de busca incorporou termos controlados e palavras-chave pertinentes aos componentes da estratégia PCC, combinados com os operadores *booleanos* AND e OR. A abordagem de pesquisa, abrangendo todas as palavras-chave e termos de indexação identificados, foi devidamente adaptada para cada fonte de informação consultada, como demonstrado na Figura 1 a seguir:

**Figura 1-:** Estratégia de busca por base de dados. Campina Grande, PB, Brasil, 2024

BDENF	(ti:(aleitamento materno)) OR (aleitamento) OR (amamentação) OR (alimentação ao peito) AND (alta do hospital) AND (cuidados de enfermagem) OR (cuidados primários de enfermagem) AND (atenção primária à saúde) OR (atenção básica); (ti:(aleitamento materno)) OR (amamentação) OR (alimentação ao peito) AND (estratégias) AND (cuidados de enfermagem) OR (cuidados primários de enfermagem) AND (atenção primária à saúde) OR (atenção básica)
*PubMed*	((((((((breastfeeding[Title]) OR (breast feeding[Title])) AND (patient discharge[MeSH Terms])) OR (hospitalization[MeSH Terms])) AND (nursing care[MeSH Terms])) OR (nursing care plan[MeSH Terms])) AND (health strategies[All Fields])) OR (local health strategies[All Fields])) AND (primary health care[MeSH Terms])) OR (care, primary health[MeSH Terms])
LILACS	(ti:(aleitamento materno)) OR (aleitamento) OR (amamentação) OR (alimentação ao peito) AND (alta do hospital) AND (cuidados de enfermagem) OR (cuidados primários de enfermagem) AND (atenção primária à saúde) OR (atenção básica); (ti:(aleitamento materno)) OR (amamentação) OR (alimentação ao peito) AND (estratégias) AND (cuidados de enfermagem) OR (cuidados primários de enfermagem) AND (atenção primária à saúde) OR (atenção básica)
SciELO	(ti:(aleitamento materno)) OR (amamentação) AND (cuidados de enfermagem) AND (atenção primária à saúde) OR (atenção básica)
*Scopus*	(((TITLE(Breast) AND (TITLE(Feed*) OR TITLE(Breastfeed*) OR TITLE(Breastfeeding)) AND (TITLE-ABS(Patient discharge*) OR TITLE-ABS(Infant discharge) AND (TITLE-ABS(«Nursing care») AND TITLE-ABS(Strategies) OR TITLE-ABS(«Associated Factor»))AND TITLE-ABS(Primary health care)))
CINAHL	TI ((breastfeeding OR breast-feeding OR infant feeding OR lactation OR lactating) AND (hospital discharge of the infant) AND (strategies OR resources OR methods OR techniques) AND (nursing care OR nursing interventions) AND (primary health care))
*Cochrane Library*	“breastfeeding” in Title Abstract Keyword AND “strategies” in All Text AND “primary health care” in All Text - with Publication Year from 2012 to 2024, in Trials (Word variations have been searched)
Catálogo de Teses e Dissertações - CAPES	aleitamento materno” OR “amamentação” AND “atenção primária à saúde” AND “cuidados de enfermagem”
BDTD*	“(Título:aleitamento materno E Todos os campos:alta hospitalar E Todos os campos:atenção primária à saúde)”

*BDTD = Biblioteca Digital Brasileira de Teses e Dissertações

### Fase 3 - Seleção de fontes de evidência

Os estudos obtidos na busca das bases de dados mencionadas foram transferidos para o gerenciador de referências *EndNote*, com o propósito de eliminar automaticamente estudos duplicados. Em seguida, foram exportados para o *software Rayyan*, onde se conduziram as etapas de exclusão dos estudos, começando pela análise dos títulos e resumos, seguida pela leitura completa. Essas etapas realizaram-se de maneira independente por dois revisores, sem qualquer divergência na exclusão dos artigos, o que dispensou a necessidade de consultar um terceiro revisor.

### Fase 4 – Extração de dados

A fase de extração de dados se conduziu com o intuito de organizar, analisar e interpretar as informações presentes nos estudos selecionados, conforme o objetivo estabelecido. Para esse procedimento, foi estruturado um formulário de extração de dados, abrangendo informações como: a identificação do periódico/fonte, autor, ano, país de origem do estudo, objetivos, população e amostra, design metodológico, principais desfechos do estudo e conclusão dos autores.

### Fase 5: Análise e apresentação dos dados

A análise dos dados envolveu a elaboração de uma síntese dos achados, alinhada aos objetivos da revisão, e a discussão das implicações dos resultados. A partir das informações extraídas, foi realizada uma análise descritiva e os resultados foram organizados em um quadro com as principais características dos estudos incluídos.

Os estudos foram classificados em cinco níveis de evidência: o Nível 1 abrange revisões sistemáticas e ensaios clínicos randomizados confiáveis; o Nível 2, estudos de coorte e pesquisas de “resultados”; o Nível 3, estudos de caso-controle; o Nível 4, séries de casos e estudos observacionais sem controles; e o Nível 5, opiniões de especialistas sem avaliação crítica^(15)^. Essa classificação organiza a qualidade das evidências, orientando decisões baseadas nos dados mais confiáveis.

### Aspectos éticos

Por tratar-se de uma revisão de escopo, não foi necessária a solicitação de aprovação do Comitê de Ética em Pesquisa. Declara-se, ainda, a inexistência de conflito de interesses.

## Resultados

Foram identificados 1.325 estudos (artigos, documentos, teses e dissertações), dos quais 398 foram excluídos por duplicidade, utilizando o programa *EndNote*. Após a análise de título e resumo, foram selecionados 123 estudos para leitura completa, dos quais 29 compuseram a amostra final. Para a descrição das buscas e a seleção dos estudos, utilizou-se o *Preferred Reporting Items for Systematic Review and Meta-Analyses* (PRISMA), conforme ilustrado na Figura 2, apresentada a seguir.

Nota: Adaptado conforme as diretrizes do *Preferred Reporting Items for Systematic Review and Meta-Analyses* (PRISMA-ScR)^(13)^

**Figura 2-:** Fluxograma do processo de seleção dos estudos incluídos na revisão. Campina Grande, PB, Brasil, 2024

Quanto aos continentes, os estudos estiveram distribuídos em sua maioria, na América do Sul (41,38%), seguido por América do Norte (31,03%), Ásia (13,79%), Europa (6,9%), Oceania (3,45%) e América Central (3,45%).

A caracterização dos estudos identificados é apresentada abaixo, considerando aspectos como autoria, ano e local de publicação, além do delineamento e do nível de evidência, como apresentado na Figura 3.

**Figura 3-:** Caracterização dos estudos incluídos na revisão. Campina Grande, PB, Brasil, 2024

**Artigo**	**Autor principal**	**Ano**	**País**	**Tipo de estudo**	Nível de evidência*
1	Iopp, et al.	2023	Brasil	Descritivo e transversal	2c
2	Martins, et al	2024	Brasil	Descritivo e qualitativo	2c
3	Sobral, et al.	2023	Brasil	Retrospectivo e abordagem mista	2c
4	Montoya, et al.	2020	Colômbia	Estudo descritivo, transversal	2c
5	Lojander, et al.	2024	Finlândia	Estudo correlacional não-experimental	5
6	Kwan, et al.	2021	Malásia	Revisão de escopo	4
7	Top, et al.	2022	Turquia	Estudo descritivo, transversal	2c
8	Luz, et al.	2018	Brasil	Estudo de coorte aberto e prospectivo	2b
9	Araújo, et al	2023	Brasil	Revisão integrativa	4
10	Fundação Oswaldo Cruz	2018	Brasil	Protocolo de boas práticas	5
11	Cordell, et al.	2020	EUA	Revisão sistemática	1a
12	Wood, et al.	2016	EUA	Revisão sistemática	1a
13	Santos, et al.	2012	Brasil	Revisão integrativa	4
14	Zanlorenzi GB	2022	Brasil	Estudo metodológico	5
15	Dahl L	2015	EUA	Capítulo de livro	5
16	Álvarez-Peña, et al.	2023	México	Estudo descritivo e qualitativo	2c
17	Levene, et al.	2024	Reino Unido	Revisão sistemática e meta-análise	1a
18	Laili FJ	2021	Indonésia	Revisão sistemática	2a
19	Ministério da Saúde (BR)	2015	Brasil	Manual do Ministério da Saúde do Brasil	5
20	Boersma, et al.	2017	Canadá	Protocolo de boas práticas	5
21	Parker LA	2013	EUA	Revisão integrativa	4
22	Ahmed, et al.	2016	EUA	Ensaio clínico randomizado controlado	1b
23	Düzgün, et al.	2020	Turquia	Revisão sistemática e meta-análise	1a
24	Noble, et al.	2018	EUA	Protocolo de boas práticas	5
25	Brodribb W	2018	EUA	Protocolo clínico	5
26	Hoyt-Austin, et al.	2022	EUA	Protocolo clínico	5
27	Wright A.	2022	Austrália	Protocolo de boas práticas	5
28	Rodrigues, et al.	2013	Brasil	Revisão integrativa	4
29	Ministério da Saúde (BR)	2013	Brasil	Documento governamental	5

*Classificação dos níveis de evidência segundo o *Oxford Centre for Evidence-Based Medicine* (2009)^(15)^

Sobre o delineamento metodológico, predominaram os estudos de revisão (34,48%) incluindo escopo, integrativa e sistemática, seguido por protocolos e guias (27,59%), transversal (13,79%), pesquisas transversais qualitativas (6,9%). Por fim, houve a frequência de apenas um artigo para estudo de coorte (3,45%), ensaio clínico randomizado (3,45%), estudo correlacional não experimental (3,45%), tese (3,45%) e capítulo de livro (3,45%). Quanto ao período de publicação, a maioria foi publicada entre 2018 e 2024 (68,97%), seguida pelo período entre 2012 e 2017 (31,03%).

A seguir, a Figura 4 oferece uma síntese das principais características dos estudos incluídos na revisão de escopo, destacando aspectos-chave, como objetivos, desfechos principais, conclusões e as estratégias obtidas. A apresentação detalhada dos estudos permite avaliar a relevância e a qualidade das abordagens investigadas, facilitando a análise crítica e o direcionamento das interpretações.

**Figura 4-:** Descrição dos estudos incluídos na revisão de escopo. Campina Grande, PB, Brasil, 2024

ID*	**Objetivos**	**Principais desfechos e conclusão**	**Estratégias**
A ^†^ 1	Conhecer as ações do enfermeiro no apoio ao AM ^‡^ .	Suporte reduz o desmame precoce e aborda riscos.	Orientação sobre massagem, extração de leite, cuidados para prematuros e gêmeos.
A ^†^ 2	Descrever a experiência de promoção ao AM ^‡^ em UBS ^§^ .	Houve melhoria dos indicadores de saúde. Desafios incluem a rotatividade da equipe.	Atividades em grupo, materiais informativos e apoio à equipe.
A ^†^ 3	Analisar a translactação e a relactação no retorno ao AM ^‡^ .	Eficácia de 78,6% em reversão do desmame.	Translactação, relactação, estimulação e ordenha mamária.
A ^†^ 4	Identificar as causas do abandono e o sucesso na relactação.	Relactação eficaz para prematuros, com apoio contínuo.	Combinação AM ^‡^ + suplementação e uso de relactadores.
A ^†^ 5	Analisar a exclusividade do AM ^‡^ pós-alta hospitalar.	Suporte rápido melhora o AM ^‡^ em primíparas.	Restringir suplementos, apoio digital e visitas domiciliares.
A ^†^ 6	Examinar a atividade galactagoga de plantas.	O feno-grego e o cardo-leiteiro demonstraram eficácia. Recomendam-se mais pesquisas.	Uso supervisionado de galactagogos.
A ^†^ 7	Determinar métodos para aumentar o leite materno.	Práticas tradicionais e modernas fortalecem a confiança.	Esvaziamento eficaz, abordagens culturais e treinamentos.
A ^†^ 8	Avaliar AM ^‡^ exclusivo em prematuros pós-alta.	Taxas abaixo do recomendado pela OMS ^‖^ .	Políticas públicas, atenção contínua e suporte pós-alta.
A ^†^ 9	Analisar evidências sobre a produção de leite materno.	Métodos como acupuntura e relactação são eficazes em 72,22%.	Uso de medicamentos, fitoterápicos e protocolos educativos.
A ^†^ 10	Manter a lactação e estabelecer o AM ^‡^ efetivo.	O apoio domiciliar e técnicas como translactação são essenciais.	Translactação, monitoramento do peso e ajuste da pega.
A ^†^ 11	Analisar o impacto de apoio social no AM ^‡^ .	Suporte social e intervenções educativas prolongam o AM ^‡^ .	Grupos de apoio, programas comunitários e canais de comunicação.
A ^†^ 12	Avaliar intervenções sobre AM ^‡^ .	Educação sobre o comportamento e sinais melhora a percepção de insuficiência de leite.	Abordagem educativa e sessões de autogestão.
A ^†^ 13	Conhecer práticas para AM ^‡^ em prematuros na UTI ^¶^ .	O apoio da rede profissional e familiar essencial para o sucesso.	Contato pele a pele, hidratação e terapias complementares.
A ^†^ 14	Desenvolver um protocolo de enfermagem sobre AM ^‡^ na APS**.	A capacitação contínua e o uso de tecnologias melhoram indicadores.	Educação audiovisual e digital com abordagem cultural.
A ^†^ 15	Abordar problemas na amamentação.	A extração ineficaz reduz a produção. Técnicas adequadas são fundamentais.	Bombeamento com vácuo confortável e técnicas de relaxamento.
A ^†^ 16	Compartilhar experiências sobre relactação.	Fortalece o vínculo mãe-bebê e a qualidade de vida.	Relactação com suporte técnico e emocional.
A ^†^ 17	Avaliar o impacto do relaxamento no AM ^‡^ .	O relaxamento aumenta a produção de leite e reduz a ansiedade materna.	Musicoterapia e práticas de relaxamento.
A ^†^ 18	Saber como aumentar a produção de leite.	Métodos combinados melhoram a lactação e reduzem estresse.	Massagem, acupressão, feno-grego e afirmações positivas.
A ^†^ 19	Promover AM ^‡^ com cuidado integral à saúde infantil.	O suporte familiar e a dieta equilibrada superam dificuldades.	Envolvimento da comunidade, técnicas de pega e ingestão calórica adequada.
A ^†^ 20	Apoiar a prática clínica baseada em evidências.	Ajustes garantem a transferência adequada de leite e nutrição.	Compressões mamárias e alternância na mamada.
A ^†^ 21	Analisar estratégias de expressão de leite em prematuros.	Diretrizes devem enfatizar o início precoce e a maior frequência de expressão.	Bombas adequadas e treinamento específico para mães.
A ^†^ 22	Determinar o impacto de sistema interativo no AM ^‡^ .	O monitoramento *online* melhora o AM ^‡^ em populações carentes.	Intervenções *web* e apoio de especialistas.
A ^†^ 23	Investigar o efeito da musicoterapia no AM ^‡^ .	Música reduz estresse e aumenta a produção de leite.	Incentivo à escolha musical e sessões regulares.
A ^†^ 24	Orientar cuidados pós-alta da UTI ^¶^ neonatal.	A transição eficaz exige um plano conjunto e ajustes maternos.	Monitoramento da pega, alimentação tripla e plano de amamentação.
A ^†^ 25	Criar protocolos para problemas no AM ^‡^ .	Galactagogos devem ser usados com supervisão médica.	Avaliação de hipolactação e drenagem eficaz das mamas.
A ^†^ 26	Fornecer suporte ao AM ^‡^ pós-alta.	A atenção à dor e o suporte contínuo melhoram indicadores.	Visitas domiciliares e apoio remoto de profissionais.
A ^†^ 27	Oferecer informações sobre o AM ^‡^ a profissionais.	A produção otimizada melhora a confiança e reduz a suplementação.	Mamadas noturnas, extração eficaz e descanso adequado.
A ^†^ 28	Avaliar fatores associados ao AM ^‡^ em prematuros.	A ordenha e o vínculo mãe-bebê são essenciais para prematuros.	Uso do copo, redução de estresse e educação materna.
A ^†^ 29	Promover o AM ^‡^ exclusivo e a alimentação complementar saudável.	A Estratégia Amamenta e Alimenta Brasil qualifica profissionais e reduz práticas inadequadas.	Qualificação, monitoramento e incentivo à amamentação.

*ID = Identificação da produção; ^†^A1-^†^A29 = Produções de 1-29; ^‡^AM = Aleitamento Materno; ^§^UBS = Unidade Básica de Saúde; ^‖^OMS = Organização Mundial da Saúde; ^¶^UTI = Unidade de Terapia Intensiva; **APS = Atenção Primária à Saúde

Para fins de análise, os estudos incluídos foram organizados em quatro categorias temáticas: abordagens educacionais e sociais para promoção da amamentação (31,0%), suporte familiar, comunitário e profissional no aleitamento (34,5%), técnicas para retomada da amamentação (24,1%) e intervenções complementares para otimização da produção de leite (34,5%). Ao considerar a natureza multifatorial das intervenções, um mesmo estudo pôde ser classificado em mais de uma categoria.

Na Figura 5 estão apresentadas as principais estratégias identificadas nos estudos incluídos, distribuídas conforme as quatro categorias temáticas previamente descritas. Essas estratégias contemplam ações fundamentadas em evidências e direcionadas ao suporte contínuo à amamentação.

**Figura 5–:** Estratégias para manutenção do AM identificadas na revisão de escopo. Campina Grande, PB, Brasil, 2024

Abordagens educacionais e sociais para promoção da amamentação
A2, A11: Ações educativas, suporte social, atividades em grupo e materiais informativos.
A13: Criação de um ambiente tranquilo e contato pele a pele.
A14: Protocolo de enfermagem com vídeos e ilustrações.
A1, A24: Orientação sobre pega adequada, riscos de fórmulas e bicos artificiais.
A10: Manter a pega correta e observar a coordenação entre sucção e respiração.
A5: Uso de alternativas digitais para melhorar a satisfação materna.
A22: Intervenção via *web* com monitoramento e notificações.
Suporte familiar, comunitário e profissional no aleitamento
A1, A8: Suporte multiprofissional contínuo.
A10, A12: Acompanhamento domiciliar e monitoramento do ganho de peso.
A19, A29: Apoio contínuo de profissionais da APS ^†^ .
A4, A19, A26: Suporte contínuo da família, comunidade e profissionais.
A14: Desenvolvimento de protocolo de enfermagem para manejo do aleitamento materno.
A25: Desenvolvimento de protocolos clínicos para gerenciar problemas médicos relacionados à amamentação.
Técnicas para retomada da amamentação
A3, A4: Técnicas de translactação e relactação.
A16: Estimulação frequente e contato pele a pele durante a relactação.
A15: Combinação de estimulação manual e bombeamento elétrico.
A20: Alternar os seios durante a mamada ( *switch nursing* ) para estimular o reflexo de ejeção.
A4, A28: Utilização de dispositivos de apoio ao AM (copinho, relactador).
A21: Início precoce e aumento da frequência de expressão de leite.
A21, A28: Contato pele a pele para melhorar a produção de leite.
Intervenções complementares para otimização da produção de leite
A9, A13: Massagem e acupuntura para aumentar a produção de leite.
A18: Combinação de acupressão e afirmações positivas.
A17: Intervenções de relaxamento para melhorar a lactação e bem-estar materno.
A23: Intervenção musical para otimizar a produção de leite.
A6: Uso de plantas galactagogas como feno-grego.
A7, A9: Uso de medicamentos e fitoterápicos.
A25: Uso de domperidona e metoclopramida como galactagogos.
A19, A27: Ingestão aumentada de calorias e líquidos; dieta equilibrada.

A = Produção; ^†^APS = Atenção Primária à Saúde

A promoção do AM enfrenta desafios interligados que demandam estratégias eficazes. A desinformação pode ser enfrentada com ações educativas, enquanto a confusão de bicos, associada ao uso de chupetas e mamadeiras, exige orientações sobre alternativas seguras. O estresse e a privação de sono, por sua vez, requerem redes de apoio e incentivo ao autocuidado. A hipogalactia, eventualmente relacionada a esses fatores, pode ser abordada com estímulo frequente das mamas, contato pele a pele e dispositivos adequados, aliados ao suporte técnico e emocional.

## Discussão

Com base nos resultados sistematizados, observa-se que as estratégias voltadas à promoção, ao apoio e à retomada do AM envolvem diferentes enfoques, agrupados em quatro categorias temáticas para fins de análise. A seguir, discute-se cada uma dessas categorias, à luz das evidências disponíveis e dos contextos de aplicação das intervenções.

### Técnicas para retomada da amamentação

Diversos estudos apontam a eficácia das práticas de relactação e translactação para restabelecer o AM, especialmente em casos de desmame precoce e em recém-nascidos prematuros. Com o suporte adequado, a relactação se mostrou eficaz, ajudando na produção de leite materno, principalmente em bebês com baixo peso ao nascer^(16)^. Nesse contexto, um estudo demonstrou que 78,6% das crianças retomaram a amamentação após a implementação dessas técnicas^(17)^. Além disso, alguns protocolos, como o ABM *Clinical Protocol* #12, orientam a transição de bebês prematuros da UTIN para o domicílio, promovendo o AME^(18)^, enquanto o ABM *Clinical Protocol* #9 aborda a suplementação com leite humano ou fórmulas e o uso de dispositivos auxiliares^(19)^.

Outrossim, um estudo revelou que o início precoce da expressão de leite, a frequência aumentada das sessões de extração e o contato pele a pele são fatores decisivos para otimizar a produção de leite em mães de bebês de muito baixo peso, destacando também a importância do acesso a bombas adequadas e o treinamento dos profissionais de saúde para apoiar as mães^(20)^. Nesse contexto, compressões mamárias são recomendadas para melhorar a transferência de leite. A alternância dos seios durante a mamada (*switch nursing*) também foi apontada como uma técnica eficaz para estimular múltiplas descidas de leite, garantindo uma melhor nutrição para o bebê e uma experiência mais positiva para a mãe^(21)^.

### Suporte familiar, comunitário e profissional no aleitamento

O suporte contínuo de profissionais e familiares é essencial para manter o AM, especialmente após a alta hospitalar^(22)^. Estudos realizados nos Estados Unidos mostram que intervenções educacionais e sociais podem aumentar a duração e a exclusividade da amamentação^(23)^, superando barreiras como a percepção de insuficiência de leite^(24)^. No Brasil, a educação pré-natal tem se mostrado eficaz na melhoria dos indicadores de saúde relacionados ao aleitamento^(25)^.

Documentos ministeriais ressaltam a importância do apoio multiprofissional e da participação da família e da comunidade para o sucesso da amamentação até os dois anos^(26)^. Esse suporte deve continuar após a alta hospitalar, com acompanhamento comunitário e consultas regulares^(27)^. O ABM *Clinical Protocol* #2 reforça o acompanhamento centrado na família^(28)^, e modelos de atendimento com capacitação e apoio comunitário têm mostrado bons resultados no SUS, ajudando a prevenir o desmame precoce^(29)^.

### Intervenções complementares para otimização da produção de leite

Uma revisão de estudos clínicos na Malásia avaliou o uso de plantas galactagogas, como o feno-grego e o cardo-leiteiro, que demonstraram efeitos positivos na produção de leite materno^(30)^, sendo alternativas naturais e eficazes para mães com dificuldades na lactação^(31)^. Além disso, práticas não farmacológicas, como acupuntura e musicoterapia, também mostraram potencial para aumentar a produção de leite e melhorar o bem-estar emocional das mães, apesar de resultados variáveis^(32)^. A combinação dessas intervenções, juntamente com técnicas como massagem mamária, acupressão e o uso de galactagogos, pode promover a lactação e reduzir o estresse materno^(33)^.

A acupuntura tem mostrado potencial para aumentar a produção de leite materno ao estimular hormônios como ocitocina e prolactina. Uma revisão sistemática indicou que muitas mães que passaram por sessões de acupuntura relataram aumento significativo na quantidade de leite. Além disso, essa técnica ajuda a reduzir o estresse e a dor, promovendo relaxamento, o que facilita a amamentação. Embora amplamente utilizada na Ásia, ainda faltam pesquisas rigorosas para padronizar protocolos e avaliar sua eficácia, mas as evidências sugerem que pode ser útil para mães com dificuldades na lactação^(34)^.

A musicoterapia tem mostrado efeitos positivos na promoção do AM, especialmente quando as mães ouvem música relaxante durante a expressão de leite, com sessões de 30 minutos repetidas ao menos 10 vezes, o que pode aumentar a produção láctea e reduzir o estresse^(35)^. Além disso, ela contribui para a diminuição da ansiedade e melhora a saúde mental das mães, fatores importantes para o sucesso da amamentação. Embora a evidência sobre seus efeitos no sono dos bebês e na redução do cortisol no leite seja limitada, a prática é bem aceita, sem riscos e acessível para as mães^(36)^.

### Abordagens educacionais e sociais para promoção da amamentação

Intervenções educacionais e apoio social contínuo aumentam a duração e exclusividade da amamentação, abordando causas modificáveis como estresse e falta de conhecimento sobre a produção de leite^(25)^. Atividades em grupo, materiais informativos e suporte à permanência das equipes de saúde são essenciais para o sucesso do aleitamento^(29)^, assim como contatos frequentes com as mães e suas redes de apoio^(23)^.

O uso de tecnologias digitais tem se destacado como recurso essencial no apoio à amamentação, especialmente entre populações vulneráveis, oferecendo acesso a informações e orientações em tempo real para mães com acesso limitado a serviços presenciais^(22)^. Essas ferramentas ampliam o suporte ao aleitamento, proporcionando dados de qualidade e auxílio personalizado^(32)^. Grupos de apoio *online*, canais diretos com especialistas e plataformas de monitoramento têm mostrado eficácia na resolução de desafios, prolongando a amamentação e criando um ambiente favorável^(37)^.

Além disso, a formação em enfermagem deve ser aprimorada para fortalecer o apoio ao AM, enfatizando a capacitação contínua dos profissionais. Isso envolve a utilização de recursos digitais e a integração de tecnologias inovadoras que ajudem a enfrentar desafios culturais e sociais relacionados à amamentação^(29)^.

Este estudo teve como limitações o número reduzido de pesquisas desenvolvidas especificamente na Atenção Primária à Saúde, sendo incluídas estratégias com potencial de aplicação nesse nível de atenção, considerando o papel da APS na continuidade do cuidado. Ademais, alguns artigos foram excluídos por não estarem disponíveis na íntegra. Para minimizar o viés, a pesquisa foi ampliada para várias bases de dados, incluindo protocolos e documentos relacionados. A classificação do nível de evidência não foi utilizada como critério para aceitar ou rejeitar estratégias, pois não fazia parte do escopo metodológico. Dessa forma, recomendam-se novas pesquisas para explorar os determinantes identificados e sua aplicabilidade nos desfechos reais.

## Conclusão

A revisão de escopo identificou diferentes estratégias para a manutenção e retomada do AM após a internação hospitalar, destacando ações educativas, suporte multiprofissional e familiar, intervenções baseadas em evidências, terapias complementares e uso de tecnologias para ampliar o acesso à informação e ao apoio. Ressalta-se, ainda, a importância de ambientes favoráveis e de diretrizes que orientem a prática profissional no contexto domiciliar.

Recomenda-se o aprofundamento de pesquisas sobre a efetividade dessas intervenções, com foco no uso de tecnologias no seguimento pós-alta, na padronização de técnicas e na avaliação dos impactos na continuidade do AM. Tais resultados podem subsidiar políticas públicas e qualificar o cuidado materno-infantil na Atenção Primária à Saúde.

Destaca-se, por fim, a necessidade de ações interdisciplinares que ampliem o acesso e a resolutividade do cuidado no pós-alta, integrando essas medidas ao cotidiano assistencial e fortalecendo a promoção e sustentação do aleitamento materno.

## Data Availability

Todos os dados gerados ou analisados durante este estudo estão incluídos neste artigo publicado.

# Translation: Estrategias para la lactancia materna después de la hospitalización del lactante: revisión de alcance*

**DOI:** 10.1590/1518-8345.7800.4673

**Authors:** Socorro Alana Ramalho Rocha, Eliane Cristina da Silva Buck, Cintia Bezerra Almeida Costa, Smalyanna Sgren da Costa Andrade

**Affiliations:** 1 Faculdade de Enfermagem e Medicina Nova Esperança, João Pessoa, PB, Brasil Faculdade de Enfermagem e Medicina Nova Esperança PB João Pessoa Brasil; 2 Universidade Federal da Paraíba, Departamento de Enfermagem, João Pessoa, PB, Brasil Universidade Federal da Paraíba Departamento de Enfermagem PB João Pessoa Brasil

**Keywords:** Lactancia Materna, Alta del Paciente, Atención de Enfermería, Estrategias de Salud, Atención Primaria de Salud, Lactancia

## Abstract

mapear estrategias dirigidas a promover y continuar con la lactancia materna después de la hospitalización del lactante.

revisión de alcance que sigue las recomendaciones del *Joanna Briggs Institute Reviewer’s Manual.* La búsqueda se realizó en siete bases de datos y en repositorios de tesis y disertaciones. La selección de estudios fue realizada mediante una aplicación *web* por dos revisores independientes y a ciegas. El análisis de datos se realizó de forma descriptiva.

se obtuvieron 1.325 publicaciones, de las cuales 29 fueron incluidas en el estudio. Se identificaron las siguientes estrategias de salud asociadas a la lactancia materna: educación y orientación, apoyo y seguimiento, intervenciones clínicas y técnicas, terapias complementarias, tecnología e innovación, ambiente y condiciones físicas, nutrición e hidratación, protocolo y guías.

la evidencia ratifica que las estrategias multifacéticas son esenciales después del alta para fortalecer la autogestión y continuar con la lactancia materna fuera del entorno hospitalario. La Atención Primaria de Salud puede desempeñar un papel importante en la continuidad de la atención.

## Destacados

**(1)** Con técnicas basadas en evidencia se puede restablecer la lactancia materna. **(2)** El apoyo familiar y profesional es fundamental para continuar con la lactancia materna. **(3)** La educación y el apoyo social aumentan la duración y la exclusividad de la lactancia materna. **(4)** Las tecnologías amplían el acceso a la información y el apoyo a la lactancia materna. **(5)** Las intervenciones complementarias ayudan a optimizar la producción de leche.

## Introducción

Los recién nacidos y los bebés son más vulnerables a las enfermedades debido a la inmadurez inmunológica. La lactancia materna exclusiva (LME) es esencial para fortalecer la inmunidad y prevenir enfermedades, pero la separación de la madre durante la hospitalización, asociada a la condición de prematuridad, puede provocar el destete precoz, que afecta el desarrollo infantil y aumenta el riesgo de hospitalización^(1)^.

La presencia de la madre durante la hospitalización es crucial, y se recomiendan prácticas como el contacto piel con piel, el alojamiento conjunto y el apoyo educativo para la lactancia materna (LM), como lo demuestra una revisión sistemática^(2)^. Además, es fundamental estimular la succión, con ayuda de profesionales capacitados y protocolos adecuados^(3)^. Al respecto, la Academia de Medicina de la Lactancia Materna (*Academy of Breastfeeding Medicine* - ABM) desarrolla recomendaciones para una lactancia materna exitosa, como el protocolo clínico N.º 35, que apoya la lactancia materna durante la hospitalización^(4)^.

Por lo tanto, el uso de enfoques innovadores y herramientas de salud puede fortalecer el aprendizaje de las mujeres al promover la lactancia materna y fomentar conductas preventivas^(5)^. Las tecnologías involucradas, que incluyen intervenciones gerenciales, educativas y asistenciales, son fundamentales para la promoción, prevención y atención de la salud^(6)^. En el área materno-infantil, los materiales didáctico-pedagógicos ayudan a orientar sobre la LM, fomentan la autonomía de la familia y contribuyen a la autoeficacia de la madre^(7)^.

Un ensayo clínico con 112 mujeres embarazadas reveló que el uso del álbum ilustrado en el grupo intervención aumentó significativamente las tasas de LME, la probabilidad de éxito fue dos veces mayor que la del grupo control (p < 0,001)^(8)^. A su vez, el estudio sobre el desarrollo y la aplicación del folleto: “*Toda mulher é capaz de amamentar!*” (“¡Todas las mujeres pueden amamantar!”) demostró que dicha tecnología educativa promovió el empoderamiento de las madres y el intercambio de conocimiento con las puérperas y sus acompañantes, y fortaleció la autoeficacia para amamantar^(9)^.

Los enfermeros desempeñan un papel esencial en la promoción y el apoyo a la lactancia materna, al brindar orientación técnica y apoyo emocional, especialmente en la Atención Primaria de Salud (APS), que coordina los cuidados en la Red de Atención^(10)^. Centrado en la salud infantil, el abordaje prioriza los vínculos, las vivencias de las madres y el uso de tecnologías blandas^(11)^, empoderando a mujeres, familias y comunidades en el entorno domiciliario. Esta atención integrada puede reducir las admisiones hospitalarias y fortalecer las políticas públicas^(6)^, y consolidar a la APS como un punto clave para la promoción de la lactancia materna.

Por lo tanto, el objetivo del estudio es mapear estrategias dirigidas a promover y continuar con la lactancia materna después de la hospitalización del lactante. Esta revisión se basa en la posibilidad de reunir un acervo de información que sirva de base para crear futuras tecnologías blandas-duras, dirigidas a proteger y brindar soporte básico para el seguimiento de la LM en el entorno domiciliario.

## Método

### Diseño del estudio

Esta revisión de alcance se realizó siguiendo la metodología del Instituto Joanna Briggs (JBI)^(12)^, junto con las recomendaciones de la *checklist Preferred Reporting Items for Systematic Reviews and Meta-Analyses* (PRISMA-ScR)^(13)^. El protocolo fue registrado en la plataforma *Open Science Framework* (OSF), al que se puede acceder mediante el enlace: https://doi.org/10.17605/OSF.IO/XJ374

Para realizar la revisión se consideraron los siguientes pasos: Fase 1 – Criterios de elegibilidad; Fase 2 – Fuentes de información y búsqueda bibliográfica; Fase 3 – Selección de fuentes de evidencia; Fase 4 – Extracción de datos; y Fase 5: Análisis y presentación de datos.

### Fase 1 - Criterios de elegibilidad

Para formular el objetivo del estudio se utilizó el acrónimo PCC (población, concepto y contexto): P (población) = lactantes; C (concepto) = lactancia materna; C (contexto) = post-hospitalización. La pregunta orientadora del estudio fue: ¿Cuáles son las producciones científicas sobre estrategias dirigidas a promover y continuar con la lactancia materna después de la hospitalización del lactante?

Se consideraron estudios completos sobre el tema, que abarcan diferentes diseños metodológicos, publicados y no publicados (literatura gris), sin restricciones de idioma. En cuanto al periodo de publicación, se estableció el rango de 2012 a 2024, dado que en 2012 se aprobó el objetivo mundial relacionado con la nutrición infantil, estipulado por la OMS/OPS, por medio de la Asamblea Mundial de la Salud, a fin de promover un aumento del 50% de la tasa de lactancia materna exclusiva para el año 2025^(14)^. Este hecho fue considerado un hito político de gran impacto sobre el tema.

### Fase 2 - Fuentes de información y búsqueda bibliográfica

Para identificar estudios relevantes se consultaron las bases de datos de revistas de la Base de Datos de Enfermería (BDENF), Literatura Latinoamericana y del Caribe en Ciencias de la Salud (LILACS), a través de la plataforma de la Biblioteca Virtual en Salud (BVS), además de la *National Library of Medicine* (*PubMed*), *Scientific Electronic Library Online* (SciELO), *Cumulative Index Nursing Allied Health Literature* (CINAHL), *Cochrane Library* y *Scopus*.

Además, se realizó un relevamiento en fuentes adicionales, como *Google Scholar*, *Catálogo de Teses e Dissertações da CAPES* y *Biblioteca Digital Brasileira de Teses e Dissertações* (BDTD). Además, se realizó una búsqueda manual en la lista de referencias de los estudios seleccionados.

Con el fin de obtener una visión integral del estado actual del conocimiento, se desarrolló una estrategia de búsqueda de cuatro pasos, siguiendo las directrices del manual del JBI para revisiones de alcance^(12)^. En primer lugar, se realizó una búsqueda inicial en las bases de datos BDENF y *PubMed*, utilizando términos DeCS-MeSH y términos índices, para examinar los títulos y resúmenes de los artículos encontrados e identificar términos que se puede adicionar a la estrategia de búsqueda. A continuación, se adaptó la táctica de investigación a las otras bases de datos, considerando las características de cada una de ellas.

Posteriormente, se realizó una búsqueda en fuentes de literatura gris para complementar los hallazgos de las producciones indexadas en las bases de datos, y favorecer la incorporación de información relacionada con el manejo técnico en LM. Además, se realizó una búsqueda manual en la lista de referencias de los estudios seleccionados para cerciorarse de que la información estuviera actualizada.

El proceso de búsqueda incorporó términos controlados y palabras clave relevantes para los componentes de la estrategia PCC, combinados con los operadores *booleanos* AND y OR. El enfoque de investigación, que abarca todas las palabras clave y términos de indexación identificados, se adaptó debidamente a cada fuente de información consultada, como se muestra en la Figura 1 a continuación:

**Figura 1:** – Estrategia de búsqueda en la base de datos. Campina Grande, PB, Brasil, 2024

BDENF	(ti:(aleitamento materno)) OR (aleitamento) OR (amamentação) OR (alimentação ao peito) AND (alta do hospital) AND (cuidados de enfermagem) OR (cuidados primários de enfermagem) AND (atenção primária à saúde) OR (atenção básica); (ti:(aleitamento materno)) OR (amamentação) OR (alimentação ao peito) AND (estratégias) AND (cuidados de enfermagem) OR (cuidados primários de enfermagem) AND (atenção primária à saúde) OR (atenção básica)
*PubMed*	((((((((breastfeeding[Title]) OR (breast feeding[Title])) AND (patient discharge[MeSH Terms])) OR (hospitalization[MeSH Terms])) AND (nursing care[MeSH Terms])) OR (nursing care plan[MeSH Terms])) AND (health strategies[All Fields])) OR (local health strategies[All Fields])) AND (primary health care[MeSH Terms])) OR (care, primary health[MeSH Terms])
LILACS	(ti:(aleitamento materno)) OR (aleitamento) OR (amamentação) OR (alimentação ao peito) AND (alta do hospital) AND (cuidados de enfermagem) OR (cuidados primários de enfermagem) AND (atenção primária à saúde) OR (atenção básica); (ti:(aleitamento materno)) OR (amamentação) OR (alimentação ao peito) AND (estratégias) AND (cuidados de enfermagem) OR (cuidados primários de enfermagem) AND (atenção primária à saúde) OR (atenção básica)
SciELO	(ti:(aleitamento materno)) OR (amamentação) AND (cuidados de enfermagem) AND (atenção primária à saúde) OR (atenção básica)
*Scopus*	(((TITLE(Breast) AND (TITLE(Feed*) OR TITLE(Breastfeed*) OR TITLE(Breastfeeding)) AND (TITLE-ABS(Patient discharge*) OR TITLE-ABS(Infant discharge) AND (TITLE-ABS(«Nursing care») AND TITLE-ABS(Strategies) OR TITLE-ABS(«Associated Factor»))AND TITLE-ABS(Primary health care)))
CINAHL	TI ((breastfeeding OR breast-feeding OR infant feeding OR lactation OR lactating) AND (hospital discharge of the infant) AND (strategies OR resources OR methods OR techniques) AND (nursing care OR nursing interventions) AND (primary health care))
*Cochrane Library*	“breastfeeding” in Title Abstract Keyword AND “strategies” in All Text AND “primary health care” in All Text - with Publication Year from 2012 to 2024, in Trials (Word variations have been searched)
*Catálogo de Teses e Dissertações - CAPES*	aleitamento materno” OR “amamentação” AND “atenção primária à saúde” AND “cuidados de enfermagem”
BDTD*	“(Título:aleitamento materno E Todos os campos:alta hospitalar E Todos os campos:atenção primária à saúde)”

*BDTD = *Biblioteca Digital Brasileira de Teses e Dissertações*

### Fase 3 - Selección de fuentes de evidencia

Los estudios obtenidos en la búsqueda en las bases de datos mencionadas fueron ingresados al gestor de referencias *EndNote*, a fin de eliminar automáticamente los estudios duplicados. Luego fueron exportados al *software Rayyan*, donde se realizaron los pasos de exclusión de estudios, que comenzó con el análisis de títulos y resúmenes, seguido de la lectura completa. Estos pasos fueron realizados de forma independiente por dos revisores y, dado que no hubo desacuerdo en la exclusión de artículos, no fue necesario consultar a un tercer revisor.

### Fase 4 – Extracción de datos

La fase de extracción de datos se realizó con el objetivo de organizar, analizar e interpretar la información presente en los estudios seleccionados, de acuerdo con el objetivo establecido. Para este procedimiento se estructuró una ficha de extracción de datos, que incluye información como: identificación de la revista/fuente, autor, año, país de origen del estudio, objetivos, población y muestra, diseño metodológico, principales resultados del estudio y conclusión de los autores.

### Fase 5: Análisis y presentación de datos

El análisis de datos implicó elaborar una síntesis de los hallazgos, que respondían al objetivo de la revisión, y llevar a cabo la discusión de las implicancias de los resultados. Con base en la información extraída, se realizó un análisis descriptivo y se organizaron los resultados en un cuadro con las principales características de los estudios incluidos.

Los estudios se clasificaron en cinco niveles de evidencia: el nivel 1 incluye revisiones sistemáticas confiables y ensayos clínicos aleatorizados; Nivel 2, estudios de cohortes y investigación de “resultados”; Nivel 3, estudios de casos y controles; Nivel 4, series de casos y estudios observacionales sin controles; y Nivel 5, opiniones de expertos sin evaluación crítica^(15)^. Esta clasificación organiza la calidad de la evidencia y orienta decisiones basadas en los datos más confiables.

### Aspectos éticos

Como se trató de una revisión de alcance, no fue necesario solicitar la aprobación del Comité de Ética de Investigación. También se declara que no hay conflicto de intereses.

## Resultados

Se identificaron 1.325 estudios (artículos, documentos, tesis y disertaciones), de los cuales 398 fueron excluidos por estar duplicados, utilizando el programa *EndNote*. Luego de analizar el título y el resumen, se seleccionaron 123 estudios para lectura completa, de los cuales 29 constituyeron la muestra final. Para la descripción de la búsqueda y selección de estudios se utilizó el *Preferred Reporting Items for Systematic Review and Meta-Analyses* (PRISMA), como se ilustra en la Figura 2, que se presenta a continuación.

**Figura 2-:** Diagrama de flujo del proceso de selección de los estudios incluidos en la revisión. Campina Grande, PB, Brasil, 2024

Nota: Adaptado según las directrices del *Preferred Reporting Items for Systematic Review and Meta-Analyses* (PRISMA-ScR)^(13)^

En cuanto a los continentes, los estudios se distribuyeron mayoritariamente en América del Sur (41,38%), seguido de América del Norte (31,03%), Asia (13,79%), Europa (6,9%), Oceanía (3,45%) y América Central (3,45%).

En la Figura 3 se presenta la caracterización de los estudios identificados, considerando aspectos como autoría, año y lugar de publicación, además del diseño y nivel de evidencia.

**Figura 3-:** Caracterización de los estudios incluidos en la revisión. Campina Grande, PB, Brasil, 2024

**Artículo**	**Autor principal**	**Año**	**País**	**Tipo de estudio**	Nivel de evidencia*
1	Iopp, et al.	2023	Brasil	Descriptivo y transversal	2c
2	Martins, et al.	2024	Brasil	Descriptivo y cualitativo	2c
3	Sobral, et al.	2023	Brasil	Enfoque retrospectivo y mixto	2c
4	Montoya, et al.	2020	Colombia	Estudio descriptivo y transversal	2c
5	Lojander, et al.	2024	Finlandia	Estudio correlacional no experimental	5
6	Kwan, et al.	2021	Malasia	Revisión de alcance	4
7	Top, et al.	2022	Turquía	Estudio descriptivo y transversal	2c
8	Luz, et al.	2018	Brasil	Estudio de cohorte abierto y prospectivo	2b
9	Araújo, et al.	2023	Brasil	Revisión integradora	4
10	*Fundação Oswaldo Cruz*	2018	Brasil	Protocolo de buenas prácticas	5
11	Cordell, et al.	2020	EE. UU.	Revisión sistemática	1a
12	Wood, et al.	2016	EE. UU.	Revisión sistemática	1a
13	Santos, et al.	2012	Brasil	Revisión integradora	4
14	Zanlorenzi GB	2022	Brasil	Estudio metodológico	5
15	Dahl L	2015	EE. UU.	Capítulo de libro	5
16	Álvarez-Peña, et al.	2023	México	Estudio descriptivo y cualitativo	2c
17	Levene, et al.	2024	Reino Unido	Revisión sistemática y metaanálisis	1a
18	Laili FJ	2021	Indonesia	Revisión sistemática	2a
19	*Ministério da Saúde* (BR)	2015	Brasil	Manual del Ministerio de Salud de Brasil	5
20	Boersma, et al.	2017	Canadá	Protocolo de buenas prácticas	5
21	Parker LA	2013	EE. UU.	Revisión integradora	4
22	Ahmed, et al.	2016	EE. UU.	Ensayo clínico controlado aleatorizado	1b
23	Düzgün, et al.	2020	Turquía	Revisión sistemática y metaanálisis	1a
24	Noble, et al.	2018	EE. UU.	Protocolo de buenas prácticas	5
25	Brodribb W	2018	EE. UU.	Protocolo clínico	5
26	Hoyt-Austin, et al.	2022	EE. UU.	Protocolo clínico	5
27	Wright A.	2022	Australia	Protocolo de buenas prácticas	5
28	Rodrigues, et al.	2013	Brasil	Revisión integradora	4
29	*Ministério da Saúde* (BR)	2013	Brasil	Documento gubernamental	5

*Clasificación de los niveles de evidencia según el *Oxford Centre for Evidence-Based Medicine* (2009)^(15)^

En lo que respecta al diseño metodológico predominaron los estudios de revisión (34,48%) de alcance, integradora y sistemática, seguidos de protocolos y guías (27,59%), transversales (13,79%), investigaciones transversales cualitativas (6,9%). Por último, se observó que la frecuencia fue de solo un artículo para estudio de cohorte (3,45%), ensayo clínico aleatorizado (3,45%), estudio correlacional no experimental (3,45%), tesis (3,45%) y capítulo de libro (3,45%). En cuanto al periodo de publicación, la mayoría se publicaron entre 2018 y 2024 (68,97%), seguido del periodo comprendido entre 2012 y 2017 (31,03%).

En la Figura 4 se presenta una síntesis de las principales características de los estudios incluidos en la revisión de alcance, y destaca aspectos clave como: objetivos, principales resultados, conclusiones y estrategias obtenidas. La presentación detallada de los estudios permite evaluar la pertinencia y calidad de los enfoques investigados, lo que facilita el análisis crítico y la orientación de las interpretaciones.

**Figura 4-:** Descripción de los estudios incluidos en la revisión de alcance. Campina Grande, PB, Brasil, 2024

ID*	**Objetivos**	**Resultados principales y conclusión**	**Estrategias**
A ^†^ 1	Conocer las acciones del enfermero para apoyar la LM ^‡^ .	El apoyo reduce el destete temprano y aborda los riesgos.	Brindar orientación sobre masajes, extracción de leche, cuidados para bebés prematuros y gemelos.
A ^†^ 2	Describir la experiencia de promover la LM ^‡^ en UBS ^§^ .	Hubo una mejora en los indicadores de salud. Los desafíos incluyen la rotación de personal.	Actividades grupales, material informativo y apoyo al equipo.
A ^†^ 3	Analizar la LM ^‡^ por sonda y relactación para retomar la LM ^‡^ .	78,6% de efectividad en la reversión del destete.	LM ^‡^ por sonda, relactación, estimulación mamaria y extracción de leche materna.
A ^†^ 4	Identificar las causas del abandono y el éxito en la relactación.	Relactación efectiva para bebés prematuros, con apoyo continuo.	Combinación LM ^‡^ + suplementación y uso de relactadores.
A ^†^ 5	Analizar la exclusividad de la LM ^‡^ después del alta hospitalaria.	El apoyo rápido mejora la LM ^‡^ en mujeres primíparas.	Restringir suplementos, apoyo digital y visitas domiciliarias.
A ^†^ 6	Examinar la actividad galactagoga de las plantas.	Se ha demostrado que el fenogreco y el cardo mariano son eficaces. Se recomienda realizar más investigaciones.	Uso supervisado de galactagogos.
A ^†^ 7	Determinar métodos para aumentar la leche materna.	Las prácticas tradicionales y modernas fortalecen la confianza.	Vaciado efectivo, enfoques culturales y capacitación.
A ^†^ 8	Evaluar la LM ^‡^ exclusiva en lactantes prematuros después del alta hospitalaria.	Tasas inferiores a las recomendadas por la OMS ^‖^ .	Políticas públicas, atención continua y apoyo después del alta hospitalaria.
A ^†^ 9	Analizar la evidencia sobre la producción de leche materna.	Métodos como la acupuntura y la relactación son efectivos en el 72,22%.	Uso de medicamentos, medicina herbal y protocolos educativos.
A ^†^ 10	Continuar con la lactancia y establecer una LM ^‡^ efectiva.	El apoyo domiciliario y técnicas como la LM ^‡^ por sonda son esenciales.	LM ^‡^ por sonda, control de peso y ajuste del agarre.
A ^†^ 11	Analizar el impacto del apoyo social en la LM ^‡^ .	El apoyo social y las intervenciones educativas prolongan la LM ^‡^ .	Grupos de apoyo, programas comunitarios y canales de comunicación.
A ^†^ 12	Evaluar intervenciones sobre LM ^‡^ .	La educación sobre el comportamiento y las señales mejora la conciencia sobre el suministro insuficiente de leche.	Enfoque educativo y sesiones de autogestión.
A ^†^ 13	Conocer las prácticas de LM ^‡^ en prematuros en UCI ^¶^ .	El apoyo de la red profesional y familiar es fundamental para el éxito.	Contacto piel con piel, hidratación y terapias complementarias.
A ^†^ 14	Desarrollar un protocolo de enfermería sobre LM ^‡^ en la APS**.	La formación continua y el uso de tecnologías mejoran los indicadores.	Educación audiovisual y digital con enfoque cultural.
A ^†^ 15	Abordar los problemas de la LM ^‡^ .	La extracción ineficiente reduce la producción. Las técnicas adecuadas son esenciales.	Técnicas de bombeo de vacío cómodas y relajación.
A ^†^ 16	Compartir experiencias sobre la relactación.	Fortalece el vínculo madre-bebé y la calidad de vida.	Relactación con apoyo técnico y emocional.
A ^†^ 17	Evaluar el impacto de la relajación en la LM ^‡^ .	La relajación aumenta la producción de leche y reduce la ansiedad de la madre.	Musicoterapia y prácticas de relajación.
A ^†^ 18	Saber cómo aumentar la producción de leche.	Los métodos combinados mejoran la lactancia y reducen el estrés.	Masaje, acupresión, fenogreco y afirmaciones positivas.
A ^†^ 19	Promover la LM ^‡^ con atención integral a la salud infantil.	El apoyo familiar y una dieta equilibrada superan las dificultades.	Participación de la comunidad, técnicas de agarre e ingesta calórica adecuada.
A ^†^ 20	Apoyar la práctica clínica basada en evidencia.	Los ajustes garantizan una adecuada transferencia de leche y la nutrición.	Compresiones mamarias y alternar el pecho durante la lactancia.
A ^†^ 21	Analizar estrategias de extracción de leche en lactantes prematuros.	Las directrices deben enfatizar el inicio temprano y una mayor frecuencia de extracción.	Bombas de leche adecuadas y capacitación específica para las madres.
A ^†^ 22	Determinar el impacto del sistema interactivo en la LM ^‡^ .	El seguimiento *online* mejora la LM ^‡^ en poblaciones vulnerables.	Intervenciones a través de la *web* y apoyo de especialistas.
A ^†^ 23	Investigar el efecto de la musicoterapia en la LM ^‡^ .	La música reduce el estrés y aumenta la producción de leche.	Fomentar la elección musical y sesiones regulares.
A ^†^ 24	Brindar orientación sobre los cuidados después del alta de la UCI neonatal.	Una transición efectiva requiere un plan conjunto y ajustes por parte de la madre.	Monitoreo del agarre, triple alimentación y plan de lactancia materna.
A ^†^ 25	Crear protocolos para problemas en la LM ^‡^ .	Los galactagogos deben utilizarse bajo supervisión médica.	Evaluación de hipolactación y drenaje mamario efectivo.
A ^†^ 26	Brindar apoyo a la LM ^‡^ después del alta hospitalaria.	La atención al dolor y el apoyo continuo mejoran los indicadores.	Visitas a domicilio y soporte remoto de profesionales.
A ^†^ 27	Proporcionar información sobre LM ^‡^ a los profesionales.	La producción optimizada mejora la confianza y reduce la suplementación.	Alimentación nocturna, extracción eficaz y descanso adecuado.
A ^†^ 28	Evaluar los factores asociados a la LM ^‡^ en prematuros.	La extracción de leche materna y el vínculo madre-bebé son esenciales para los bebés prematuros.	Uso de vaso, reducción del estrés y educación materna.
A ^†^ 29	Promover la LM ^‡^ exclusiva y la alimentación complementaria saludable.	La *Estratégia* *Amamenta e Alimenta Brasil* capacita a profesionales y reduce prácticas inadecuadas.	Cualificación, seguimiento e incentivo de la lactancia materna.

*ID = Identificación de producción; ^†^A1-^†^A29 = Producciones de 1-29; ^‡^LM = Lactancia materna; ^§^UBS = Unidad Básica de Salud; ^‖^OMS = Organización Mundial de la Salud; ^¶^UCI = Unidad de Cuidados Intensivos; **APS = Atención Primaria de Salud

Para el análisis, los estudios incluidos se organizaron en cuatro categorías temáticas: enfoques educativos y sociales para promover la lactancia materna (31,0%), apoyo familiar, comunitario y profesional a la lactancia materna (34,5%), técnicas para reanudar la lactancia materna (24,1%) e intervenciones complementarias para optimizar la producción de leche (34,5%). Considerando la naturaleza multifactorial de las intervenciones, un mismo estudio puede clasificarse en más de una categoría.

A continuación, se presentan las principales estrategias identificadas en los estudios incluidos, distribuidas según las cuatro categorías temáticas previamente descritas. Estas estrategias incluyen acciones basadas en evidencia dirigidas al apoyo continuo a la lactancia materna, como se ilustra en la Figura 5:

**Figura 5-:** Estrategias para continuar con la LM identificadas en la revisión de alcance. Campina Grande, PB, Brasil, 2024

Enfoques educativos y sociales para promover la lactancia materna
A2, A11: Acciones educativas, apoyo social, actividades grupales y material informativo.
A13: Crear un ambiente tranquilo y contacto piel con piel.
A14: Protocolo de enfermería con vídeos e ilustraciones.
A1, A24: Orientación sobre agarre adecuado, riesgos de las fórmulas y tetinas artificiales.
A10: Mantener el agarre correcto y observar la coordinación entre succión y respiración.
A5: Uso de alternativas digitales para mejorar la satisfacción de la madre.
A22: Intervención a través de la *web* con seguimiento y notificaciones.
Apoyo familiar, comunitario y profesional a la lactancia materna
A1, A8: Apoyo multidisciplinario continuo.
A10, A12: Seguimiento domiciliario y control del aumento de peso.
A19, A29: Apoyo continuo de los profesionales de la APS*.
A4, A19, A26: Apoyo continuo de la familia, la comunidad y los profesionales.
A14: Desarrollo de un protocolo de enfermería para el manejo de la lactancia materna.
A25: Desarrollo de protocolos clínicos para el manejo de problemas médicos relacionados con la lactancia materna.
Técnicas para reanudar la lactancia materna
A3, A4: Técnicas de lactancia materna por sonda y relactación.
A16: Estimulación frecuente y contacto piel con piel durante la relactación.
A15: Combinación de estimulación manual y bombeo eléctrico.
A20: Cambio de pecho durante la lactancia ( *switch nursing* ) para estimular el reflejo de eyección.
A4, A28: Uso de dispositivos de apoyo a la lactancia (vaso, relactador).
A21: Inicio temprano y aumento de la frecuencia de extracción de leche.
A21, A28: Contacto piel con piel para mejorar la producción de leche.
Intervenciones complementarias para optimizar la producción de leche
A9, A13: Masaje y acupuntura para aumentar la producción de leche.
A18: Combinación de acupresión y afirmaciones positivas.
A17: Intervenciones de relajación para mejorar la lactancia y el bienestar de la madre.
A23: Intervención musical para optimizar la producción de leche.
A6: Uso de plantas galactagogas como el fenogreco.
A7, A9: Uso de medicamentos y medicina herbal.
A25: Uso de domperidona y metoclopramida como galactagogos.
A19, A27: Aumento de la ingesta de calorías y líquidos; alimentación equilibrada.

A = Producción; *APS = Atención Primaria de Salud

La promoción de la LM enfrenta desafíos interconectados que demandan estrategias efectivas. La desinformación se puede abordar con acciones educativas, mientras que la confusión de pezón, asociada al uso de chupetes y biberones, requiere orientación sobre alternativas seguras. El estrés y la falta de sueño, a su vez, requieren redes de apoyo y estímulo para el autocuidado. La hipogalactia, posiblemente relacionada con estos factores, se puede abordar con estimulación mamaria frecuente, contacto piel con piel y dispositivos apropiados, junto con apoyo técnico y emocional.

## Discusión

A partir de los resultados sistematizados, se observa que las estrategias dirigidas a promover, apoyar y retomar la LM incluyen distintos enfoques, que fueron agrupados en cuatro categorías temáticas para el análisis. A continuación, se analiza cada una de estas categorías, en función de la evidencia disponible y de los contextos en que se aplican las intervenciones.

### Técnicas para reanudar la lactancia materna

Varios estudios indican que las prácticas de relactación y LM por sonda son efectivas para restablecer la LM, especialmente en casos de destete precoz y en recién nacidos prematuros. Se ha demostrado que, con el apoyo adecuado, la relactación es eficaz y contribuye a la producción de leche materna, especialmente en bebés con bajo peso al nacer^(16)^. De hecho, un estudio demostró que el 78,6% de los niños retomaron la lactancia materna después de implementar esas técnicas^(17)^. Además, algunos protocolos, como el ABM *Clinical Protocol* #12, brindan orientación sobre la transición de los bebés prematuros de la UCIN al hogar y promueven la lactancia materna exclusiva^(18)^, mientras que el ABM *Clinical Protocol* #9 aborda la suplementación con leche materna o fórmula y el uso de dispositivos^(19)^.

Además, un estudio reveló que el inicio temprano de la extracción de leche, el aumento de la frecuencia de las sesiones de extracción y el contacto piel con piel son factores decisivos para optimizar la producción de leche en madres de bebés de muy bajo peso al nacer, ambién destaca la importancia del acceso a extractores adecuados y la capacitación de los profesionales de la salud para apoyar a las madres^(20)^. En este caso, se recomiendan las compresiones mamarias para mejorar la transferencia de leche. Cambiar el pecho (*switch nursing*) durante la lactancia también se identificó como una técnica eficaz para estimular múltiples liberaciones de leche, lo que garantiza una mejor nutrición para el bebé y una experiencia más positiva para la madre^(21)^.

### Apoyo familiar, comunitario y profesional a la lactancia materna

El apoyo continuo de los profesionales y los familiares es fundamental para continuar con la lactancia materna, especialmente después del alta hospitalaria^(22)^. Estudios realizados en Estados Unidos revelan que las intervenciones educativas y sociales pueden aumentar la duración y exclusividad de la lactancia materna^(23)^, superando barreras como la percepción de suministro insuficiente de leche^(24)^. En Brasil, la educación prenatal demostró ser eficaz para mejorar los indicadores de salud relacionados con la lactancia materna^(25)^.

Los documentos ministeriales destacan la importancia del apoyo multidisciplinario y la participación familiar y comunitaria para una lactancia materna exitosa hasta los dos años de edad^(26)^. Este apoyo debe continuar después del alta hospitalaria, con seguimiento comunitario y consultas regulares^(27)^. El ABM *Clinical Protocol* #2 destaca la atención centrada en la familia^(28)^, y los modelos de atención con capacitación y apoyo comunitario demostraron que dan buenos resultados en el SUS, y ayudan a prevenir el destete precoz^(29)^.

### Intervenciones complementarias para optimizar la producción de leche

Una revisión de estudios clínicos en Malasia evaluó el uso de plantas galactagogas, como el fenogreco y el cardo mariano, y reveló que las mismas tenían efectos positivos en la producción de leche materna^(30)^, y que son alternativas naturales y efectivas para las madres con problemas en la lactancia^(31)^. Además, las prácticas no farmacológicas, como la acupuntura y la musicoterapia, también demostraron que tenían potencial para aumentar la producción de leche y mejorar el bienestar emocional de las madres, a pesar de que los resultados fueron variables^(32)^. La combinación de estas intervenciones, junto con técnicas como el masaje mamario, la acupresión y el uso de galactagogos, puede promover la lactancia y reducir el estrés de la madre^(33)^.

Se ha demostrado que la acupuntura tiene potencial para aumentar la producción de leche materna al estimular hormonas como la oxitocina y la prolactina. Una revisión sistemática indicó que muchas madres que se sometieron a sesiones de acupuntura informaron un aumento significativo de la producción de leche. Además, esta técnica ayuda a reducir el estrés y el dolor, favoreciendo la relajación, lo que facilita la lactancia. Si bien se utiliza ampliamente en Asia, aún faltan investigaciones rigurosas para estandarizar los protocolos y evaluar su eficacia, pero la evidencia sugiere que puede ser útil para las madres con problemas en la lactancia^(34)^.

La musicoterapia demostró tener efectos positivos para promover la lactancia materna, especialmente cuando las madres escuchan música relajante mientras realizan la extracción de leche, con sesiones de 30 minutos repetidas al menos 10 veces, lo que puede aumentar la producción de leche y reducir el estrés^(35)^. Además, ayuda a reducir la ansiedad y mejora la salud mental de las madres, que son factores importantes para una lactancia materna exitosa. Aunque la evidencia sobre los efectos en el sueño de los bebés y la reducción del cortisol en la leche es limitada, la práctica tiene buena aceptación, no presenta riesgos y es accesible para las madres^(36)^.

### Enfoques educativos y sociales para promover la lactancia materna

Las intervenciones educativas y el apoyo social continuo aumentan la duración y la exclusividad de la lactancia materna al abordar causas modificables como el estrés y la falta de conocimiento sobre la producción de leche^(25)^. Las actividades grupales, el material informativo y el apoyo a la permanencia de los equipos de salud son esenciales para el éxito de la lactancia materna^(29)^, al igual que el contacto frecuente con las madres y sus redes de apoyo^(23)^.

El uso de tecnologías digitales se destaca como un recurso esencial para apoyar la lactancia materna, especialmente en las poblaciones vulnerables, dado que brinda acceso a información y orientación en tiempo real para madres con acceso limitado a los servicios presenciales^(22)^. Dichas herramientas amplían el apoyo a la lactancia materna proporcionando datos de calidad y asistencia personalizada^(32)^. Los grupos de apoyo *online*, los canales directos con expertos y las plataformas de seguimiento demostraron que son eficaces para resolver desafíos, prolongar la lactancia materna y crear un entorno favorable^(37)^.

Por otro lado, es necesario mejorar la formación de enfermería para fortalecer el apoyo a la LM, fundamentalmente la educación continua de los profesionales. Esto implica el uso de recursos digitales y la incorporación de tecnologías innovadoras que contribuyan a enfrentar los desafíos culturales y sociales relacionados con la lactancia materna^(29)^.

Se consideran limitaciones del estudio el pequeño número de investigaciones desarrolladas específicamente en Atención Primaria de Salud, que incluyen estrategias que pueden se implementadas en ese nivel de atención, considerando el papel de la APS en la continuidad de la atención. Además, se excluyeron algunos artículos porque no estaban disponibles para lectura completa. Para minimizar el sesgo, la búsqueda se amplió a múltiples bases de datos, e incluyó protocolos y documentos relacionados. La clasificación del nivel de evidencia no se utilizó como criterio para aceptar o rechazar estrategias, ya que no forma parte del alcance metodológico. Por lo tanto, se recomienda realizar más investigaciones para explorar los determinantes identificados y su aplicabilidad a resultados reales.

## Conclusión

La revisión de alcance identificó diferentes estrategias para continuar y reanudar la lactancia materna después del ingreso hospitalario, entre las que se destacaron acciones educativas, apoyo multidisciplinario y familiar, intervenciones basadas en evidencia, terapias complementarias y uso de tecnologías para ampliar el acceso a la información y al apoyo. También se destaca la importancia de entornos favorables y pautas que orienten la práctica profesional en el entorno domiciliario.

Se recomienda realizar más investigaciones sobre la efectividad de dichas intervenciones, que se centren en el uso de tecnologías para el seguimiento posterior al alta hospitalaria, la estandarización de técnicas y la evaluación del impacto sobre la continuidad de la LM. Estos resultados pueden contribuir a la creación de políticas públicas y a cualificar la atención materno infantil en la Atención Primaria de Salud.

Por último, destacamos que es necesario implementar acciones interdisciplinarias que amplíen el acceso y la resolutividad de los cuidados después del alta hospitalaria, e integren esas medidas a los cuidados diarios y fortalezcan la promoción y el apoyo a la lactancia materna.

## Data Availability

Todos los datos generados o analizados durante este estudio están incluidos en este artículo publicado.
